# Questions concerning the role of amyloid-β in the definition, aetiology and diagnosis of Alzheimer’s disease

**DOI:** 10.1007/s00401-018-1918-8

**Published:** 2018-10-22

**Authors:** Gary P. Morris, Ian A. Clark, Bryce Vissel

**Affiliations:** 1 Centre for Neuroscience and Regenerative Medicine, Faculty of Science, University of Technology Sydney, Sydney, NSW Australia; 2 St Vincent’s Centre for Applied Medical Research (AMR), St Vincent’s Hospital Sydney Limited, Darlinghurst, Sydney, Australia; 3 Biomedical Sciences and Biochemistry, Research School of Biology, Australian National University, Canberra, ACT Australia

## Abstract

The dominant hypothesis of Alzheimer’s disease (AD) aetiology, the neuropathological guidelines for diagnosing AD and the majority of high-profile therapeutic efforts, in both research and in clinical practice, have been built around one possible causal factor, amyloid-β (Aβ). However, the causal link between Aβ and AD remains unproven. Here, in the context of a detailed assessment of historical and contemporary studies, we raise critical questions regarding the role of Aβ in the definition, diagnosis and aetiology of AD. We illustrate that a holistic view of the available data does not support an unequivocal conclusion that Aβ has a central or unique role in AD. Instead, the data suggest alternative views of AD aetiology are potentially valid, at this time. We propose that an unbiased way forward for the field, beyond the current Aβ-centric approach, without excluding a role for Aβ, is required to come to an accurate understanding of AD dementia and, ultimately, an effective treatment.

**Electronic supplementary material:**

The online version of this article (10.1007/s00401-018-1918-8) contains supplementary material, which is available to authorized users.

Our major goal must be the prevention of AD, and achievement of this goal requires that we first understand its cause.

Katzman [138]

## Background

The recent National Institute on Aging and Alzheimer’s Association (NIA-AA) Research Framework is an interesting document [119]. Clearly, the words “a biological definition” in the title implies searching for biomarkers with an essential and defining functional role in the pathogenesis of Alzheimer’s disease (AD). Yet the text might read more as a commitment to keeping both the amyloid hypothesis and the amyloid removal concept of AD treatment in the forefront of the research agenda, rather than as the new approach the field awaits.

One cannot ignore the data supporting a possible role of amyloid-β (Aβ), nor rule out a plausible clinical rationale for removing it, but the present data does not prove Aβ has, or will have, a central role in AD nosology, aetiology or diagnosis. On the contrary, many would question whether, in the face of the extraordinary accumulation of inconsistencies and controversies surrounding the amyloid hypothesis [179], and an accumulation of evidence that supports alternative views of aetiology [39, 43, 59, 176, 204, 220, 272], Aβ pathology should still be highly regarded as a biomarker that defines AD as a unique neurodegenerative disorder.

In fact, the literature indicates neither amyloid plaques, nor neurofibrillary tangle (NFT) deposits, are unique to AD, as suggested in their abstract [119], since these pathologies are also paired in other neurodegenerative states, such as post-stroke syndromes [268], Parkinson’s disease (PD) [203], traumatic brain injury (TBI) [140], HIV-dementia [288], Lewy body dementia [54] and lead poisoning [158]. Indeed, Alois Alzheimer and his contemporaries noted similarities in the clinical and pathological presentations of syphilitic dementia and AD [171]. These examples demonstrate how these conditions appear to be on the same pathophysiological spectrum, as distinct from Aβ pathology being unique to an ‘Alzheimer’s continuum’, as suggested [119].

There are at least nine modifiable risk factors for AD, all of which may reduce disease risk independently of Aβ and/or tau pathology [161]. As noted [161], focussing on unique features in the whole person, rather than a single feature, is crucial to successfully altering the course of disease. We argue that this principle also applies as the AD field looks to define, and pharmacologically target, biological features of this disease. The rest of this text summarises how AD research was led into this Aβ-dominated cul-de-sac, and suggests ways out of it.

## The present definition, hypothetical models of aetiology and diagnostic criteria of AD may need reform

A lack of success of promising therapeutics for AD [65, 71, 107, 231] has recently been reinforced by the departure of a discouraged Pfizer from the field. As ever, there is some hope in current trials [56]. Most notably, a recent comment from Biogen and Eisai hinted at promising Phase II results with an anti-Aβ drug [72], albeit with important caveats. Here, we debate the strengths and weaknesses of the experimental evidence supporting current therapeutic efforts and discuss whether they are too heavily reliant on what may be a flawed approach to AD nosology, aetiology and diagnosis.

In particular, we discuss an insufficiently reflected point: that the current consensus neuropathological diagnostic strategy for AD is based on no more than a working hypothesis of disease aetiology, underlined by two long-held, but unproven, assumptions (Box 1). We will illustrate that neither the historical, clinical, or preclinical records unequivocally endorse the absolute validity of these assumptions (Box 1), or the amyloid hypothesis more generally. It follows that the record cannot currently support recently proposed research guidelines for the specific identification of preclinical and prodromal states of AD, which are based on an extension of these assumptions [2, 255]. If the assumptions underlying the current predominant approach are indeed wrong, an overhaul is urgently required. Our discussion will focus on clinical AD research. We and others have reviewed the many continuing inconsistencies and controversies in preclinical studies [38, 39, 60, 179, 204, 220, 249, 272].

As will be seen, we do not suggest Aβ has no role in AD. Nevertheless, just as the effectiveness of H2 receptor antagonists and proton pump inhibitors led researchers astray on the centrality of stomach acid to stomach ulcers [165], current evidence for a role of Aβ in AD, or potential positive results with anti-Aβ agents in humans, does not and will not necessarily point to Aβ as the central cause or accurate prognostic of AD dementia. The broader point is that this debate is now essential to reach a more accurate understanding of AD.

## Box 1: Long-held assumptions supporting the hypothetical current consensus diagnostic guidelines for Alzheimer’s disease

### Assumption 1

Aβ and tau pathologies are, combined, a specific marker of AD dementia with Aβ pathology upstream of tau and other AD-associated pathologies.

### Assumption 2

AD is a single homogenous disorder in which individuals with early-onset familial or early-onset sporadic AD (onset at < 65 years of age) and late-onset familial or late-onset sporadic AD (onset at > 65 years of age) have the same disease.

## The current neuropathological diagnosis of AD propagates a hypothesis of disease aetiology

### Initial clinico-neuropathological diagnostic guidelines for AD were based on a hypothesis of aetiology

The nosology of tuberculosis, once based on the presence of tubercles, evolved when *Mycobacterium tuberculosis* was discovered as causative by Robert Koch (reviewed in [83]). AD research has similarly seen many ground-breaking discoveries in the past 30 years, but the diagnostic guidelines and hypothetical framework of pathogenesis supporting these guidelines (the amyloid hypothesis) remain fixed in a perception of aetiology first elaborated, in earnest, during the 1980s.

**Fig. 1:**
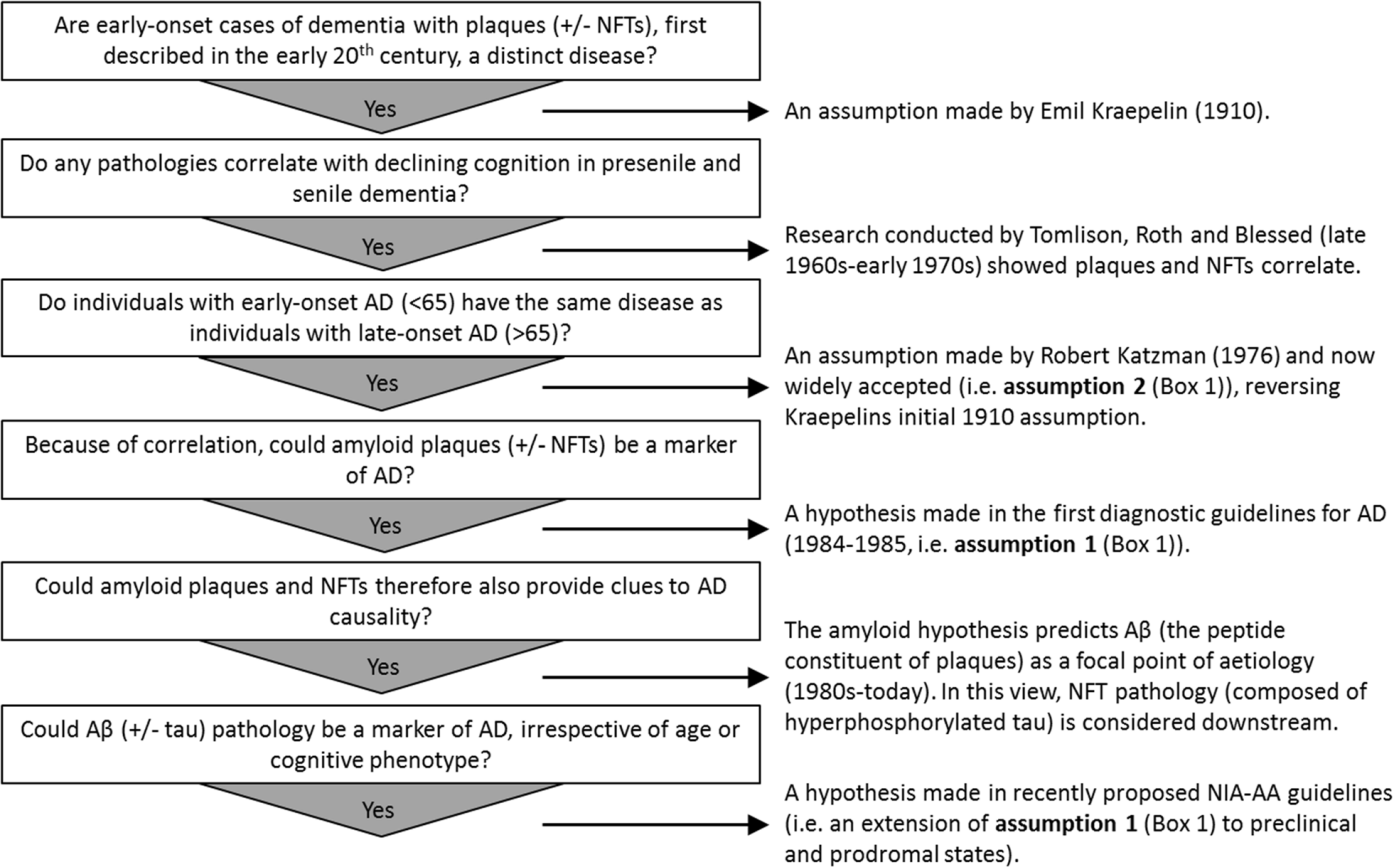
A chronological sequence of questions, assumptions and findings leading to the current Aβ-centric ‘consensus’ Alzheimer’s disease neuropathological diagnostic guidelines. Major observations, findings and assumptions have been distilled into the current ‘consensus’ diagnostic guidelines for AD. In brief, these guidelines are derived from the hypothesis that the relative levels of amyloid plaques and NFTs in specific brain regions differentiates AD dementia from normal cognitive aging. Combined with the amyloid hypothesis they predict Aβ and  tau pathology to be a specific marker of AD at symptomatic and possibly preclinical and prodromal stages, with Aβ considered causative of tau pathology (and other AD-associated pathologies). *NFTs* neurofibrillary tangles, *Aβ*  amyloid-β

In the late 1960s, Tomlinson, Roth and Blessed undertook studies suggesting numerical scores of amyloid plaques and NFTs, two brain ‘lesions’ long associated with age and dementia (Fig. 1), correlated to scores of dementia [23, 222, 269, 270]. This encouraged the idea that quantifying these lesions could be diagnostically useful to distinguish normal aging and dementia (Assumption 1, Box 1). This idea subsequently became a core feature of the first neuropathological diagnostic guidelines for AD, published in the 1980s [143, 169] (see Supplementary Material 1.1 for important extended historical context on the development of AD nosology).

A second influential change was a 1976 editorial by Robert Katzman. Building on an earlier opinion from Martin Roth (discussed in [228]), he argued that aggressive presenile dementia (i.e. early-onset AD dementia beginning at < 65 years of age, at the time labelled ‘Alzheimer’s disease’ in accordance with Emil Kraepelin’s original 1910 definition [147]) was the same disease as senile dementia (i.e. late-onset AD dementia beginning at > 65 years of age [137]). We later (Question 5) discuss whether Katzman’s view is still relevant in contemporary AD literature.

### The clinico-neuropathological diagnosis of AD remains based on a hypothesis of aetiology

The definition of AD as a cognitive disorder with amyloid plaques and NFTs essentially became the assumed truth in clinical practice and has driven research for decades. This concept drove the elucidation of the peptide constituent of the amyloid plaques (Aβ) [91, 92, 167] and of pathogenic mutations and duplications in the genes for the Aβ precursor protein, amyloid precursor protein (APP) and mutations in proteins involved in the enzymatic processing of APP into various peptides (including Aβ), presenilin 1 and 2 (PSEN1/2), in some familial AD cases [131].

Although NFTs were revealed to be composed of hyperphosphorylated tau protein [114] over this same period, identifying genetic links to *APP* and its processing enzymes drew attention firmly toward Aβ as a likely upstream cause of AD. From this focus evolved the ‘amyloid (or Aβ) (cascade) hypothesis’ (as well as a later iteration, the ‘Aβ oligomer hypothesis’ [52]), predicting that accumulated, aggregated or uncleared Aβ peptides, forming both soluble Aβ oligomers and insoluble amyloid plaques, are central to the onset and progression of AD. Indeed, as late as 2016 this concept was still regarded as *“*…the most extensively validated and compelling therapeutic target in this disease*”* [244].

Many clinico-neuropathological studies followed, as did research with genetically modified mouse models expressing familial AD-linked *APP* and *PSEN* mutations, and intense in vitro and in vivo effort assessing the synaptic and neuro ‘toxicity’ of Aβ. All this added fuel to the notion of Aβ centrality [179] in autosomal dominant AD and by inference, sporadic AD. Thus, the amyloid hypothesis became a major pillar of the fundamental assumptions contained in the first diagnostic guidelines for AD (Box 1) [143].

The notion that Aβ pathology defines AD has remained largely intact through each successive update to the diagnostic criteria [28, 108, 109, 143, 170, 172]. Until now, a diagnosis of ‘typical’ AD dementia is first clinical, defined by the presence of a hippocampal amnestic syndrome, with or without other cognitive and behavioural changes [68, 289]. This is corroborated, after obvious clinical exclusion, by in vivo or end-stage quantification of cerebral Aβ pathology, adjusted for age.

Although NFT counts are also still an essential part of the neuropathological diagnosis of AD (e.g. Braak staging of NFT pathology [68, 108]), the amyloid hypothesis predicts they and other disease-associated pathologies, including synapse degeneration, hippocampal atrophy and neuroinflammation, are downstream of Aβ pathology and less disease specific [119, 189, 236]. Therefore, if an individual presents with the clinical symptoms of AD dementia without cerebral Aβ pathology, the current view posits that individual simply does not have AD. This is a ‘consensus’ view broadly shared by both the NIA-AA diagnostic guidelines (2011–2018) [108, 119, 170, 175] and the International Work Group (IWG) criteria (2007–2014) [67, 68], which has been passed down from the 1980s guidelines [143, 169] and subsequent updates to them [28, 109, 172]. The amyloid hypothesis continues to support these guidelines, being firmly stated as the favoured hypothesis of AD aetiology by a recently commissioned NIA-AA workgroup [119].

### A litany of data leads to questions regarding the robustness of the assumption Aβ pathology is disease defining

The emergence of AD diagnostic criteria in the 1980s, in conjunction with the amyloid hypothesis, meant that, over the ensuing decades, ‘Aβ pathology’ has essentially become synonymous with ‘Alzheimer’s’. In practice, irrefutably proving the amyloid hypothesis or, more accurately, rejecting the null hypothesis that Aβ is not causally linked to AD dementia, has not yet occurred. Thus, *the current neuropathological diagnosis of AD dementia is in fact a diagnosis of a working hypothesis of disease aetiology* (for historical comments regarding the formation of the initial diagnostic guidelines in 1984/1985 see [144]). Additionally, as we discuss throughout, a litany of data has consistently emerged to question the robustness of the Aβ-centric view of AD dementia, leading to a loss of confidence in the amyloid hypothesis by many researchers [14, 38, 40, 43, 148, 185, 204, 253, 272].

All this matters because the strategy used to diagnose disease has ethical, societal and financial consequences through determining treatment strategies and research funding decisions, and moulding public opinion and health policies [216]. Increasingly, therefore, decisions based on the amyloid hypothesis are not to be taken lightly. In this context, very recent recommendations, published in 2016 [69] and 2018 [119] for predicting disease in preclinical (‘at-risk’) stages, using in vivo measurements of putative AD biomarkers, concern us.

## The extension of hypothetical diagnostic guidelines to preclinical and prodromal states

### The push to extend hypothetical diagnostic criteria for AD to preclinical and prodromal phases may be premature whilst the assumptions underlying the criteria remain unproven

In 2011 recommendations were published for identifying prodromal (‘mild cognitive impairment (MCI) due to AD [2]’) and preclinical (‘AD-pathophysiological process [255]’) stages of AD. Subsequently, proposals that these stages could be identified using in vivo ‘biomarker’ evidence of Aβ and tau pathology, irrespective of cognitive changes, have emerged.

The recent NIA-AA commissioned workgroup consolidated these proposals, and spoke of an ‘Alzheimer’s continuum’ [119]. This was defined as: *“*…individuals with biomarker designation of either AD or Alzheimer’s pathologic change*”*, wherein “biomarker designation of AD*”* refers to in vivo evidence of Aβ and tau pathology and “Alzheimer’s pathologic change*”* refers to in vivo evidence of Aβ pathology alone (with normal tau biomarkers). In this system, evidence of abnormal tau and/or neurodegeneration biomarkers, in the absence of Aβ pathology, are defined as "non-AD pathologic change". Importantly, the IWG have also proposed guidelines for the identification of these states with the use of biomarkers [69], but are less definitive than the NIA-AA workgroup on the primacy of Aβ measurements. They still allow for the possibility that those with evidence of tau pathology, in the absence of amyloid, are also at-risk for AD dementia [69]. As noted [119], the two advisory bodies are, however, harmonised on the concept of using the label ‘Alzheimer’s’ when Aβ and tau pathology are found, irrespective of the cognitive diagnosis.

Collectively therefore, these proposals appear to argue for the terms ‘Alzheimer’s continuum’, ‘Alzheimer’s Disease’ or ‘at-risk for Alzheimer’s’ being used only when Aβ (and, in the case of the IWG, tau) pathology is detected, *irrespective of a clinical diagnosis or age*. In turn, without such evidence, it is proposed the label ‘Alzheimer’s’ should not be applied.

Although these recommendations could be seen as merely a subtle extension of the neuropathological diagnostic principles that Aβ and tau pathology are the definitive neuropathological ‘proof’ of AD, they are in fact a radical departure from the traditional use of the label ‘Alzheimer’s’ only when the AD cognitive phenotype is identified. Indeed, the publication by the workgroup runs the risk of formalising the idea that AD cannot be hypothetically explained without accounting for the presence of Aβ and tau pathology, as encapsulated in the following statement:

We emphasise though that A and T proteinopathies define AD as a unique disease among the many that can lead to dementia. As a consequence, disease models where A and T are not in the primary causal pathway must provide a mechanistic explanation for the development of both of these diagnostic proteinopathies, as well as neurodegeneration and clinical symptoms. [119]

This statement presents two major problems. First, it ignores contradictory literature on the absolute validity of Assumptions 1 and 2 (Box 1), which we discuss below in Questions 1–5. Second, it ignores equally valid alternate hypotheses of disease aetiology that do not require an Aβ (and/or tau) basis. We therefore view the statement as an inaccurate representation of the current state of AD research.

We do not see the problem lying with the hypothesis that Aβ and tau biomarkers could predict disease—it is a valid and testable idea. Instead, the crux of the problem is that this statement perpetuates the idea *“*A and T proteinopathies*” define* AD dementia *as* a priori *fact*, when this remains uncertain. Potentially more worrying is that in labelling *“*A and T proteinopathies*”* under the Alzheimer’s name in the absence of the cognitive phenotype, the inference is made that effective treatments for AD might now be defined by their ability to treat *“*A and T proteinopathies*”,* whether or not there is proven clinical benefit of such treatments.

Granted, the NIA-AA workgroup acknowledged disease causation may be independent of Aβ and tau, and went to considerable lengths to stress many clinicians and researchers do not necessarily wish to adopt proposed nomenclature (noting also that it is premature to extend these criteria to the clinic). However, busy clinicians and the public at large may not appreciate this subtle point. Instead, the take-away summary for most is likely to be that AD is to be defined and thus understood through an Aβ lens. Certainly, in the absence of other readily available biological markers of AD for clinicians, Aβ pathology may be a useful marker for AD dementia *risk*. However, as we discuss below, the literature suggests the causality of AD dementia is more complex than can be accounted for by the amyloid hypothesis.

Hence, these guidelines present a hypothetical idea, that in vivo measurements of Aβ (± tau) pathology can accurately predict disease, built upon another hypothetical idea, that Aβ and tau pathology mark a specific clinical disorder (AD dementia) and therefore provide clues to aetiology. In removing the safeguard of the clinical AD diagnosis, these recommendations can be viewed as an attempt to bypass many long-held concerns regarding the validity of these hypotheses. In the following we raise key questions testing Assumptions 1 and 2 (Box 1), illustrating there is still significant discrepant evidence to address before concluding the presence of Aβ pathology is definitive for AD or aetiologically significant. If these assumptions are ultimately proven incorrect, the push to defining disease on their bases presymptomatically is running the risk of sending research into aetiology down the wrong path.

## Question 1: Do cognitively normal individuals exhibit Aβ and tau pathology?

### Answer: Pathological levels of Aβ and tau are present in cognitively normal individuals

In 1997 the unusual case of Sister Mary was introduced [250]. As part of the seminal longitudinal epidemiological ‘Nun Study’, Sister Mary was described as a ‘gold standard’ for successful aging, owing to her high cognitive test scores at the advanced age of 101. Intriguingly, despite this, upon autopsy it was revealed her brain contained abundant amyloid plaques and NFTs, satisfying the Khachaturian criteria for AD [143], but not the Consortium to Establish a Registry for Alzheimer’s Disease (CERAD) criteria [172]. Sister Mary therefore provided a conundrum: her amyloid plaques satisfied one of the neuropathological guidelines for AD, despite the fact she had no evidence of cognitive dysfunction.

Sister Mary is by no means unique, but representative of a group of individuals known variously as ‘high pathology controls’ (HPCs) [164], ‘preclinical AD’ [210], ‘asymptomatic AD’ [219] or ‘non-demented but with AD pathology’ [233]. These individuals, despite being dementia free, can have amyloid plaque and NFT counts as high as those seen in individuals with mild cognitive impairment (MCI) or dementia [111, 112, 201]. Such cases, known since at least as early as 1933 [19, 270], are far from rare: one-third of the Nun Study cohort reached neuropathological criteria for AD, despite being cognitively intact at their last assessment before death [128, 251]. Other large-scale epidemiological studies have shown the same pattern [17, 192, 233, 235]. Collectively, up to 40% of cognitively normal individuals may reach some level of neuropathological criteria for AD [209], although this figure is dependent on age (possibly increasing from 10 to 40% between ages 50–90) and *APOE4* gene status [129].

It has been contended that end-stage NFT pathology (Braak stage VI) does not exist without some evidence of cognitive impairment [1, 188]. Nevertheless, evidence indicates as many as 20% of cognitively normal elderly exhibit neuropathological AD when restrictive diagnostic criteria for Aβ and tau pathology are applied [209]. Furthermore, although late amyloid plaque and NFT stages are more common in clinical AD cohorts than the general population, not all symptomatic cases exhibit them [233], indicating neuropathological heterogeneity in symptomatic cohorts.

More recently, end-stage neuropathological findings have been supported by in vivo evidence that up to 47% of cognitively normal individuals may have amyloid positive positron-emission tomography (PET) scans (the commonly quoted figure is ~10–30% [43, 44]) and 18% of older adults have tau PET scans reaching Braak stages III/IV [241].

### The presence of amyloid plaques in cognitively normal individuals has not yet been explained

How to make sense of this pattern? It would be imprudent to ignore the many logical explanations for these well-described paradoxical cases. Indeed, the hypothetical concepts of ‘brain reserve’ and ‘cognitive reserve’ may have merit [257]. Alternatively, the location and type of plaque present (diffuse or neuritic) may be integral to the development of disease. Otherwise, the popular amyloid ‘oligomer’ hypothesis suggests soluble amyloid species might be causal in AD, potentially mitigating the aetiological importance (and therefore presence) of insoluble species.

However, though these interpretations are valid, they all remain unproven. The concepts of ‘reserve’, for example, currently lack a neural basis [257], whilst both diffuse and neuritic plaques have been found in cognitively normal individuals [281]. The amyloid oligomer theory must also be tempered by the lack of consensus on the definition [16, 263], definitive presence and biochemical composition [211, 282] of Aβ oligomers in the brain in vivo and the questionable validity of studies purporting oligomer toxicity, in part due to use of non-physiologically relevant experimental paradigms [179]. We also recall that solanezumab, developed to remove soluble forms of Aβ, did not meet primary endpoints in two phase III studies [66]. Collectively, therefore, the presence of purported disease-specific lesions in cognitively normal individuals remains unexplained. We refer the reader to Supplementary Material 1.2 for an expanded discussion of the various interpretations stated above.

### Aβ pathology is a risk factor for AD, but does not guarantee it

Although unexplained, the evidence does suggest Aβ pathology in cognitively normal and MCI is associated with a higher likelihood of progression to MCI or AD dementia [51, 221]. Moreover, a correlation has been reported between amyloid PET positivity and subjective cognitive decline in cognitively normal elderly in some [3, 202] but not all studies [44, 45]. Others have reported amyloid positivity in cognitively normal individuals is associated with low memory scores, but not Mini Mental State Examination (MMSE) scores [130] (see [8, 182]). Furthermore, there have been recent suggestions that the relative amount of amyloid plaques (i.e. a dose-response) [22, 79, 94], or the rate of accumulation [153], rather than just amyloid ‘positive’ or ‘negative’ status, is linked to cognitive decline.

Hence, the presence of amyloid in cognitively normal individuals may be useful for predicting a risk of conversion from non-symptomatic to symptomatic stages. However, these studies merely suggest Aβ pathology to be a risk factor for dementia, not necessarily a cause, let alone the sole cause. Some studies, for instance, have shown as high as 80% a non-conversion rate of amyloid positive cognitively normal individuals to MCI or dementia 2–3 years later [280], with some individuals remaining cognitively stable for up to 6 years after follow-up [44]. Furthermore, other evidence suggests injury markers, rather than amyloid markers, are better predictors of progression from MCI to AD [276]. Collectively, these results suggest the utility of Aβ pathology *alone* to predict cognitive decline may be limited, questioning its applicability as a disease defining biomarker.

## Question 2: Are there individuals diagnosed clinically with AD, but who have no Aβ and tau pathology?

### Answer: Some individuals clinically diagnosed with AD do not have Aβ pathology

Discrepancies between the clinical phenotype and the neuropathology of disease have long been known. Alzheimer’s second case, for instance, was one of plaque-only dementia, lacking tau tangles [174]. However, it was not until formal neuropathological guidelines were in place that systematic studies shed light on how widespread these discrepancies are. Reviewing the relationship of the clinical and neuropathological diagnosis across 919 subjects at National Institute on Aging Alzheimer’s Disease Centres from 2005 to 2010, Beach demonstrated a sensitivity of diagnosis ranging from 70.9 to 87.3% and specificity of 44.3–70.8% [12]. Importantly, a substantial proportion (39%) of their ‘non-AD’ dementia diagnoses exhibited AD histopathology, a finding corroborated in other studies [195]. Other significant findings were that a number of clinical AD diagnoses had low levels of Aβ pathology and that 17 of 88 cases not meeting pathological criteria for AD were diagnosed with AD regardless [12].

Discrepancies in clinical and pathological diagnoses have been verified across multiple studies. Collectively, they report limited evidence of cerebral AD pathology in approximately 10–20% of individuals clinically diagnosed with AD [12, 245], although some studies report lower numbers [224] and others report higher. More recent studies continue to highlight disagreements. For instance, a 30% discrepancy in clinical and biomarker data was found in one of two tested cohorts using a novel blood based Aβ assay [187]. Additionally, it has been illustrated there is inter-individual heterogeneity in the content and chemical characteristics of Aβ and tau pathology in the hippocampus, even amongst patients in the same neuropathological stage [85].

Notably, as indicated by Beach, many individuals with a clinical diagnosis of AD exhibit mixed pathology (e.g. combinations of amyloid plaques, cerebral infarctions, Lewy bodies, etc.). One large-scale longitudinal study found 46% of individuals with a clinical AD diagnosis had multiple pathologies [239], a finding corroborated by others (see [214]). This extends to dementia more broadly, where > 50% can exhibit mixed pathology [238]). More recently, >230 combinations of neuropathology were observed in a cohort of >1000 aged individuals [27]. Although amyloid plaque and NFT pathology was common, it rarely occured in isolation [27]. This indicates co-morbidities are the rule rather than the exception [214, 271]. Importantly, the relative level of Aβ pathology may not differentiate Aβ-only dementia and mixed-pathology dementia cases. In one study [100], for instance, the CERAD criteria for a diagnosis of AD were satisfied in 83% of cases clinically diagnosed as Lewy body dementia. Furthermore, amyloid plaque density has been shown to reach the level required for the neuropathological diagnosis of AD in cases of early-onset dementia following TBI [139]. The implication is that Aβ biomarkers may have limited ability to selectively diagnose ‘pure’ Aβ and tau pathology-only individuals.

Collectively, the data above raises the question as to whether AD dementia can be accurately defined on the basis of Aβ neuropathology. We consider this further below.

### Some individuals clinically diagnosed with AD exhibit non-Aβ pathologies

Importantly, a portion of ‘clinically misdiagnosed’ cases (i.e. Aβ pathology-negative individuals with a clinical AD diagnosis) show non-Aβ pathologies in brain sections. These so-called ‘AD mimics’ include tangle-only dementia or argyrophilic grain disease, frontotemporal lobar degeneration, cerebrovascular disease, Lewy body dementia and hippocampal sclerosis [12].

The use of in vivo diagnostic techniques measuring other markers (e.g. NFTs, brain hypometabolism and atrophy) has corroborated these discrepancies. For example, hippocampal sclerosis of aging has been identified as an AD ‘mimic’, being present in > 20% of individuals over 85. A recent proposal suggests redefining this group as ‘cerebral age-related TDP-43 and sclerosis’ (CARTS) [190]. Furthermore, many individuals clinically diagnosed with probable AD that have no or few amyloid plaques do exhibit NFTs, a phenomenon termed primary age-related tauopathy (PART) [55].

This AD mimicry concept is supported by about 25% of cognitively normal individuals, and a similar proportion of those with mild cognitive impairment (MCI), over the age of 65, exhibiting abnormal neurodegeneration biomarkers, but normal Aβ biomarkers [122]. This entity has been termed ‘suspected non-Aβ pathology’ (SNAP) and is labelled thus irrespective of cognitive status. While these categories may or may not overlap [122, 178], their presence is telling in the present context.

Crucial questions emerge when seeking to understand the above clinical and pathological discrepancies. *One* obvious question is whether or not the neuropathological criteria used to diagnose AD are associated with the pathogenesis of the clinical condition. *Another* is whether individuals without evidence of Aβ pathology are being clinically misdiagnosed. For instance, are CARTS, PART and SNAP truly ‘non-AD’, or are they merely ‘non-amyloid.’

In one study, cognitively unimpaired individuals with SNAP were found to be indistinguishable from amyloid positive individuals by both imaging and clinical criteria, as well as risk factor assessments [146]. Furthermore, separation of PART from AD has been disputed on the grounds that no neuropathological, genetic or clinical criteria differentiate such cases from early AD [70]. These arguments, debated in references [43, 55, 70, 122], are consistent with clinical AD sometimes being independent of Aβ pathology [12]. In this context it is important to note that both Aβ and non-Aβ pathologies associated with dementia account for less than half of all late-life cognitive decline, suggesting the field has barely scratched the surface of potential causes or contributors to cognitive decline, beyond histologically visible lesions [25].

In summary, the previous two sections illustrate (1) that cohorts of cognitively normal individuals can show Aβ and tau pathologies, implying amyloid deposition is not necessarily causal; (2) that other cohorts diagnosed as ‘non-AD’ dementias can exhibit amyloid plaques and tau tangles, implying these lesions are not unique to AD, and (3) Aβ-negative cohorts diagnosed clinically as AD dementia can instead exhibit other pathologies, indicating pathogenic pathways to AD dementia unrelated to amyloid.

One could eliminate these discrepancies by ignoring the clinical diagnosis and relying solely on the presence of Aβ (± tau) pathology to define AD dementia. However, as we have discussed, the literature is not this simple. Further understanding by the field, on which rational therapy depends, must face this issue.

## Question 3: Does the spatial appearance, progression and absolute amount of amyloid plaques correlate with declining cognition more conclusively than other pathologies?

The short answer is that amyloid plaques do not correlate to dementia as well as do tau tangles or synapse pathology. Arriving at this conclusion involves discussing spatial, correlative (this section) and temporal dimensions (next section) of the question.

Although the original neuropathological guidelines for AD were built on the correlation of amyloid plaques and NFT counts to cognition, much research since has established amyloid plaques are less well correlated to the clinical and anatomical progression of AD than other pathologies, including synapse loss [266] and NFTs [184, 197]. One possible reason for the disparity between the hypothetical primacy of amyloid in disease and its relatively poor correlation to clinical decline, compared with other pathologies, could be the unreliability of the early statistical findings [188, 265]. Nevertheless, evidence since has indicated neuritic plaques correlate to declining cognition better than do diffuse plaques [188], indicating that even if NFTs correlate better, plaques counts are still useful determinants of dementia severity.

Why do NFTs correlate better than amyloid plaques? This may be due to the spatially distinct anatomical locations in which each of these pathologies arise, and then propagate. NFTs propagate fairly linearly, as denoted in the Braak staging guidelines [28, 175]. These changes possibly begin subcortically, with the first cortical appearance observable in the transentorhinal region, before spreading toward neocortical regions [30], which correlates spatially better with areas undergoing degeneration than does the propagation of amyloid plaques. Amyloid plaque deposition initially begins in polymodal association cortices and spreads toward the allocortex (for summaries see [34, 38, 110]). Additionally, the deposition of amyloid plaques plateaus in later life [121] whereas the rate of neurodegeneration accelerates [124], suggesting the clinical symptoms couple to neurodegeneration, rather than Aβ deposition.

The better correlation of NFTs justifies suggestions that therapeutically [89] and diagnostically [196] targeting tau pathology may be a better alternative to anti-Aβ approaches. There are some relevant caveats to these theories. Neuron loss in AD far exceeds the number of NFTs, suggesting they may not be causal [93, 256, 264]. Furthermore, it is well understood both amyloid plaques and NFTs are present in large numbers of the cognitively normal elderly, with NFTs almost universally present in aged individuals [55]. Indeed neurons may live for decades with tau pathology [30, 181].

How can the relatively poor spatial correlation of amyloid plaques to NFTs, synapse loss and neurodegeneration be explained in a theory maintaining amyloid is primary in aetiology? One theory, with growing support, holds that Aβ deposition may trigger prion-like seeding and propagation of tau pathology in functionally connected areas [277]. However, the spreading of aggregated tau does not necessarily require the presence of amyloid deposits [70], and tau may enhance the deposition and toxicity of Aβ [207]. Theoretically AD could begin through self-propagation of Aβ aggregates via a prion-like seeding mechanism [134, 283], followed by propagation of disease through the aforementioned mechanisms (e.g. Aβ could be the ‘trigger’ and tau the ‘bullet’ [24]). Other possible explanations are discussed in Supplementary Material 1.3.

Several caveats accompany theories seeking to explain Aβ’s role in these complex ways. Most involve Aβ pathology appearing before other AD-associated pathologies, which is not yet definitive (see next section). Additionally, much of the debate has shifted to the relationship of soluble oligomeric Aβ and tau to disease, rather than insoluble species [52, 98, 244, 256], questioning the aetiological relevance of insoluble protein deposits. As mentioned earlier, theories regarding soluble Aβ and tau await a better understanding of their nature in vivo. Otherwise, a major caveat is the lack of in vivo investigation of other possible markers of the clinical AD phenotype, beyond Aβ and tau. We discuss promising alternatives below and under Question 4.

We also note that Aβ deposition occurs in the cerebrovasculature (cerebral amyloid angiopathy (CAA)) as well as in the brain parenchyma. CAA is present in up to 85–95% of individuals with AD, with 25% of AD brains having moderate-to-severe CAA [73]. Importantly, some cortical atrophy in AD may be a consequence of CAA [9, 84], suggesting CAA may be an independent contributor to cognitive [26] and pathological alterations in AD [9], despite it often being left out of the aetiological discussion. CAA is discussed in more detail in Supplementary Material 1.3.

### What pathological markers correlate with disease beyond Aβ and tau?

In the following section (Question 4), we discuss several non-Aβ and non-tau AD biomarkers worth exploring as predictors and markers of AD dementia. We briefly note here, in the context of spatial correlative studies, that markers of synapse and neuronal pathology may provide important independent indicators of disease. For example, a study using a recently developed tau PET marker suggested glucose hypometabolism (a proxy marker of neuronal function [120]) tracks the clinical progression of disease better than tau pathology [46]. This is critical, since the clinical symptoms of AD may be coupled with changes in glucose metabolism, or the rate of neurodegeneration, rather than Aβ and tau deposition [124]. Considering neurodegeneration is a likely physical cause of cognitive decline in AD [271, 286], both preceding and paralleling it [124], this is unsurprising.

Support for this comes from a cortical atrophy signature of volume loss in the hippocampus, medial and lateral parietal cortex and temporal neocortex [127]. Furthermore, neuron loss in the hippocampus, cerebral cortex and subcortical regions, and a concomitant increase in non-neuronal cell numbers, may be a differentiating feature between those with pathology who are symptomatic and those with pathology who are not [5].

The relationship of neuronal degeneration to cognitive decline is further reflected by the strong correlation of synapse dysfunction and loss to cognitive decline [62, 184, 236, 266], with synaptic abnormalities in the hippocampus, cingulate gyrus, entorhinal cortex, temporal cortex and frontal cortex particularly relevant to clinical AD dementia [62, 106]. Although evidence shows synapse pathology can occur on both living and dead neurons [52] it remains unclear if synapse loss precedes neuronal death, or whether both pathologies have distinct pathways.

Given that learning and memory depend on synapse and neural function, it is not surprising Aβ pathology would correlate less well to cognitive decline than synaptic and neurodegenerative changes. Indeed there is appreciable consensus that AD is, *ipso facto*, a synaptic disorder, even within the amyloid hypothesis [180, 243]. Thoughts differ, however, on how this synapse pathology arises [184]. Of course, alterations in Aβ are predicted by the amyloid hypothesis to precede and likely cause synapse pathology [120]. However, it must be borne in mind synapse dysfunction usually arises from perturbation in the physiological functions of cellular and molecular components within the multicellular synapse and extracellular matrix [49, 180] (discussed further in Supplementary Material 1.3). This debate therefore clearly requires consideration of the temporal appearance of possible mechanistic drivers beyond Aβ.

## Question 4: Is the temporal appearance of Aβ pathology the first biological sign of disease onset?

### Answer: It is not yet conclusive that Aβ pathology is the first putative AD biomarker to emerge along the disease ‘continuum’

A widely held idea has been that anti-Aβ clinical trials have failed simply because treatments were commenced too late in the disease process [244]. The conceptual development of ‘preclinical [255]’ and ‘prodromal [2]’ states of AD has been a significant step forward in aiming to overcome this limitation [221]. The hunt has therefore been on to identify biomarkers capable of predicting disease development during these early phases. Much of the focus has been on Aβ, which makes sense if looking to target Aβ removal early in the disease process. However, as discussed below, this has come at the cost of meaningful focus on what other biomarkers may objectively predict cognitive decline.

The most prominently tested markers to date include CSF Aβ measurements (as proxies for cerebral Aβ deposition), cerebral PET amyloid imaging, CSF total and phosphorylated tau measurements (as proxy markers of cerebral NFTs), cerebral metabolism using FDG PET (as a proxy marker of neuronal activity) and measurements of cerebral atrophy using MRI [103]. A framework for the temporal sequence of these putative AD biomarkers has been proposed [123] and novel data are often compared to it. This hypothetical sequence predicts the appearance of Aβ pathology precedes other AD-associated pathologies. The validity of this hypothesis is assessed below.

### Is Aβ pathology the first biomarker to become abnormal in autosomal dominant AD mutation carriers?

Carriers of autosomal dominant AD mutations (in *APP*, *PSEN1* and *PSEN2*) provide a useful group to test hypothetical biomarker sequences. Nevertheless, despite suggestions these populations have provide support for hypothetical models (that amyloid biomarkers become abnormal first [119]), the raw data can be unconvincing. For instance, the case was made that increased CSF Aβ42 levels are present up to 30 years in advance of clinical onset in mutation carriers, and then begin declining 25 years prior, preceding changes in other biomarkers [11]. However, although showing a trend, CSF Aβ42 levels were not statistically different compared to non-carriers at these time points. Instead, statistically significant changes occurred just 10 years before predicted onset, temporally after statistically significant changes in CSF tau, hippocampal volume, cerebral Aβ deposition and plasma Aβ at 15 years prior. A follow-up study again found statistically significant differences in CSF Aβ42 (at 10 years prior to predicted onset) were preceded by changes in other CSF markers including tau markers and a neuronal death marker VILIP-1 [75].

There is evidence that abnormal levels of CSF Aβ precede the development of metabolic, structural and tau alterations [82]. More recently, in extending findings from an earlier observational study [18], one study illustrated changes in cerebral Aβ pathology 20 years in advance of predicted onset, preceding metabolic and structural changes in some (but not all) brain regions, beginning in the precuneus (the hippocampus was a notable exception) [95]. Intriguingly, early hypermetabolism was noted alongside Aβ deposition in the earlier study, perhaps reflecting that metabolism and Aβ deposition are related to neuronal activity [18]. Further recent work with a novel in vivo tau tracer has provided evidence NFT pathology may lag cerebral Aβ pathology in *PSEN1* mutation carriers [213]. If this finding is replicated (the validity of tau tracers are still being assessed [279]), it will provide strong evidence cerebral amyloid deposition precedes cerebral tau pathology in familial cohorts.

Collectively then, studies in autosomal dominant AD cohorts have established that Aβ, tau and neurodegeneration biomarkers significantly differ in mutation carriers many years in advance of expected AD onset. Furthermore, the evidence amyloid biomarkers go awry first (amongst those tested so far) has strengthened, despite robust conclusions being limited by a lack of statistical significance at extreme ends of the expected age of onset distribution (which, as noted [75], must be interpreted with caution due to small sample sizes), inter-individual variability [168] and a lack of longitudinal assessment of multiple biomarkers concurrently. Studies are ongoing to overcome these limitations. For instance, a recent effort [168] has provided a longitudinal intra-individual dataset of putative biomarkers, supporting that cerebral Aβ pathology is an early marker of disease, albeit also highlighting important differences compared with earlier cross-sectional studies. Important to reiterate is that although Aβ pathology may have temporal precedence, not all brain regions follow the same temporal pathological patterns [95].

We note it would not be unexpected to observe abnormal Aβ measurements early in autosomal dominant cohorts, considering such mutations are known to alter Aβ production. The majority of *PSEN1* mutations, for instance, reduce total Aβ levels compared to wild-type *PSEN1* and favour Aβ42 over Aβ40 production [260]. However, there are a number of important caveats to familial data sets: (1) not all autosomal dominant AD mutations have the same influence on Aβ production [260]; (2) hypothetical biomarker trajectory models will be subject to change once comprehensive longitudinal studies are completed (and putative AD biomarkers beyond those currently available are assessed) and (3) the extrapolation of results from these cohorts to all AD must only be done so with caution.

### It is not yet clear Aβ pathology is the first biological sign of sporadic AD onset

Studies in the much larger population at risk for sporadic AD are more difficult to interpret. Aβ pathology often seems clearly detectable many years in advance of symptom onset in sporadic AD (potentially up to 20–30 years [129]). There is also some useful evidence CSF Aβ levels become abnormal more often and likely earlier than do CSF tau or hippocampal volume [125]. However, if indeed amyloid may change up to 30 years in advance of symptoms, there is also a clear possibility (and evidence) that tau pathology arises very early in the brainstem, possibly preceding cerebral deposition of Aβ [29, 259]. Indeed, evidence suggests the appearance of NFTs precedes Aβ pathology in the vast majority of affected regions [225]. The use of novel in vivo tau selective PET tracers will shed further light on the spatial and temporal relationship of these pathologies [279] (see [126, 163] for recent examples).

Meanwhile, beyond Aβ and tau, it is impossible to conclude either are the first markers of disease onset due to the distinct lack of comprehensive investment in, and validation of, alternative possible AD biomarkers. We discuss this issue below.

### Measuring multiple putative AD markers without bias may provide more accurate predictions of disease

A common limitation in temporal studies is a lack of assessment of currently available biomarkers, longitudinally, at the same time, in the same individuals [95, 154, 162], as well as investment in validating and testing biomarkers beyond those commonly used. This does not diminish the preceding efforts, which have been incredibly arduous, but illustrates the difficulty in drawing firm conclusions from current data sets that will inevitably be highly subject to change as new information emerges (see [168] for a recent example). The value of assessing multiple biomarkers together is exemplified by evidence showing Aβ and tau pathology, alone, do not predict incipient cognitive decline 7.5 years before onset as well as a combined value of the two [221].

A recent study comprehensively illustrated the utility of assessing a diverse range of putative biomarkers and in doing so highlighted the importance of non-Aβ pathologies in disease. In analysing over 7000 brain images and > 10 putative biomarkers across > 1000 healthy and diseased subjects, Iturria-Medina et al., demonstrated that vascular dysregulation could be the earliest and strongest pathological factor associated with AD, before Aβ deposition [115], contradicting the predictions of the hypothetical late-onset AD biomarker curves [123].

Another instructive example of a precedent is clusterin. As we have related previously [47] clusterin, one of the acute phase proteins, is intimately associated with onset, progression, and severity of human AD [267]. Unfortunately, only the amyloid chaperone function of this protein was discussed, rather than its role as an acute phase protein, increased in vivo by extremely small doses of the inflammatory cytokines TNF and IL-1 [99]. Clusterin was found to be raised 10 years earlier than fibrillar Aβ deposition. The relevant gene, *CLU*, is the second highest of a list of the 15 top-rated genes linked to AD on the Alzgene web-based collection [193].

Clearly, it is becoming increasingly important to objectively predict and diagnose disease using unbiased assessments of multiple putative biomarkers [41]. Evidence for the efficacy of other non-Aβ and tau markers is discussed in the following.

### Could in vivo synaptic measures be useful for prediction and diagnosis?

Synapse loss is clearly a major correlate of cognitive decline in AD. We recognise synapse dysfunction and loss could be argued to be non-specific for AD dementia [236], since it is clearly present in other neurodegenerative conditions. However, few in vivo longitudinal studies have attempted to search for signature spatial or temporal patterns of synapse dysfunction and loss, or synaptic biomarkers than could specifically mark disease, or subsets of disease. This may be due to in vivo tools for measuring synaptic deficits having only recently become available [31, 42, 141, 150]. For a recent promising example of one attempt to separate subclasses of dementia using synaptic markers see [20]. Differentiating the mechanisms underlying synapse loss in subclasses of dementia and determining if these mechanisms drive spatially distinct synapse pathology in different dementias will be an important next step.

The potential of these novel in vivo synaptic markers has recently been illustrated, with a reduction in hippocampal density visible in AD compared to cognitively normal controls using PET imaging of synaptic vesicle glycoprotein 2A (SV2A) [42]. Furthermore, CSF measurements of neurogranin, a proxy measure of synaptic loss, may predict progression from prodromal states to AD. It appears better correlated with tau, rather than amyloid [141]. Some evidence exists for synaptic alterations occurring before the appearance of Aβ deposition [80, 254], but it cannot be ruled out soluble forms of Aβ may be driving this pathology.

Considering synapse dysfunction is a strong correlate of disease, continuing to investigate the temporal and spatial progression of synapse pathology in vivo, relative to other pathologies, is a pressing priority for future research. Such studies could profoundly alter the understanding of disease; we may learn, for example, that amyloid deposition, perhaps in functionally connected areas, is a correlative marker of synapse dysfunction and loss, not the cause.

### Validating and utilising putative AD biomarkers beyond Aβ and tau pathology may assist with disease prediction and provide novel therapeutic targets

Synapses aside, it is not difficult to hypothesise Aβ pathology is a sometimes secondary factor to myriad upstream triggers. It has been demonstrated that physical, age-related and genetic perturbations might exacerbate Aβ deposition. Vascular damage [86], oxidative stress [191] and APOE4 [160] are examples. It is well established, for instance, diffuse amyloid plaques develop within hours of TBI [132] and plaque density years following TBI can reach the level required for a definite AD diagnosis [139], in spatially similar patterns to those seen in AD [242]. It is logical to consider that if there are upstream triggers of Aβ pathology (which may also trigger Aβ-independent cognitive dysfunction in AD) then significant effort should be directed towards understanding the spatial, correlative and temporal patterns of the cellular and molecular biomarkers related to these upstream triggers.

Other biomarkers under development concern mitochondrial dysfunction [81], neuronal injury (visinin-like protein-1 [142]) and axonal injury (neurofilament light [292]), amongst others. In particular, studies using in vivo markers of neuroinflammation, a possible mechanistic driver underlying many disease-associated risk factors [50], have already elucidated significant findings. The PET tracer ^11^C-deuterium-l-deprenyl (DED), a putative marker of astrocyte activation, has been shown to correlate with Aβ deposition decades before symptom onset, suggesting a very early activation of astrocytes in AD that may either drive, or be driven by, Aβ pathology [240]. Furthermore, one study illustrated that an early phase of microglial activation, detected using a translocator protein 18 kDa (TSPO) tracer, was associated with a small upregulation of Aβ pathology in vivo, but could be independent of Aβ and hence triggered by other factors [77]. Other promising examples of neuroinflammatory biomarkers are discussed in Supplementary Material 1.4.

### Bacterial, viruses, fungi and other microbial infiltration may be upstream triggers of AD and associated pathologies

There is a long history of investigation into a connection between various microbes and AD. Research links bacteria [74], fungi [205] and viruses [218], supporting theories that AD is potentially caused by infectious agents [101, 117]. There is a well-documented history, for instance, establishing clinical and pathological similarities in syphilitic dementia, caused by *Treponema pallidum*, to AD dementia, dating back to Alzheimer’s time [171].

More recently, a study has shown a population of patients with herpes simplex virus infections had a 2.56-fold increased risk of dementia. Remarkably, when comparing those treated with anti-herpetic medication to those not, the risk of dementia in these patients was reduced by 90.8% [273]. A published commentary is available [116]. When regarding these studies, one should be mindful of research showing Aβ may be an anti-microbial peptide [96] and therefore potentially acting to combat infiltrating infectious agents.

### Analyses of possible disease biomarkers should be conducted without preconceptions about temporal ordering

In summary, there are many risk factors for AD, all of which may contribute to disease through numerous cellular and molecular mechanisms, independent of, or in combination with Aβ and tau. The research reviewed above reveals a present lack of longitudinal in vivo exploration of promising avenues, beyond Aβ and tau. While we await such data it is crucial that no a priori decision is made to lump untested putative biomarkers within pre-existing, but unproven, conceptions of temporal ordering. Instead, more balanced hypotheses of temporal biomarker profiles are required, acknowledging the importance of many other factors to disease initiation and progression. A recent effort by Tse and Herrup [272] provides a holistic example.

## Question 5: How widely applicable are findings from autosomal dominant mutation carriers to sporadic AD?

### Answer: Autosomal dominant AD may represent a different disease to sporadic AD

Although further studies are clearly indicated, the fact remains that neither the clinician, the neuropathologist, nor the electron microscopist can distinguish between the two disorders, except by the age of the patient.

Katzman [137]

### Theories on the aetiology of autosomal dominant and sporadic AD are often grouped under the same banner, but this might not be correct

An important turning point for the field was the widespread acceptance of the assumption early-onset and late-onset AD were one and the same [137] (Assumption 2, Box 1). The story following this period is well known. It was expected for some time a familial component to early-onset AD was likely to exist (a later analysis of Alzheimer’s second case suggested a familial predisposition [145]). Autosomal dominant AD mutations were eventually identified in the *APP* gene on chromosome 21. A second AD locus on chromosome 14 was found in genes encoding PSEN1 and PSEN2. Mutations (and duplications, in the case of *APP*) in these genes were summarily linked to altered Aβ metabolism. In contrast to mutations in precursor genes for other AD-associated pathologies, such as tau [188], they have become gold-standard evidence of the amyloid hypothesis.

The impact of these discoveries has been profound. Many observational and therapeutic clinical efforts focus on cohorts harbouring these mutations [10] and preclinical models of disease are designed primarily by expressing these mutations in mice [234]. However, the relevance of extrapolating data garnered from observational and therapeutic studies with autosomal dominant AD carriers to sporadic AD has become less tenable over time. Their influence is at odds with their extreme rarity and although phenotypic similarities exist, so do known differences, apart from age-of-onset alone, as originally assumed [137]. Additionally, the vast array of genetic risk factors for AD, beyond autosomal dominant mutations, casts doubts on the idea all AD pathogenesis can be explained by the amyloid hypothesis. In light of this, we next discuss the validity of Assumption 2 (Box 1).

### The genetic causes and risk factors for familial and sporadic AD are largely unknown

It is important to emphasise the extreme rarity of currently known autosomal dominant AD mutations (in *APP*, *PSEN1* and *PSEN2*) and the lack of understanding of the majority of familial AD. Early-onset AD is variably reported to represent some 1–10% of all AD [33, 294], and generally thought to be familial (possibly 10% autosomal dominant and 90% autosomal recessive [287]). However, up to 95% of early-onset AD cases remain genetically unexplained [33]. Clearly, much of the genetics of AD remains unresolved (see Supplementary Material 1.5 for extended statistics on the heritability of AD).

In fact, far from being simple and linear, as the amyloid hypothesis predicts, the genetics of AD are highly complex. Causal mutations can be autosomal dominant, but these could either be inherited or arise de novo in both early-onset AD and late-onset AD [155]. Mutations could be recessive or germline/somatic and expressed in a mosaic pattern [33], as has been illustrated with Trisomy 21 [208]. Furthermore, evidence is gathering for epigenetic contributions to disease [232].

### The aetiology of AD dementia in autosomal dominant mutation carriers may be different to the aetiology of sporadic AD

Regardless of the frequency of known autosomal dominant AD mutations, two unresolved questions remain: (1) what is the exact aetiology within these cohorts and (2) how relevant is their aetiology to AD more broadly? In regard to question (1), we have previously pointed out *APP*, *PSEN1* and *PSEN2* mutations will have numerous effects on full-length APP processing and APP function, as well as effects on functions of a range of other proteins beyond APP [179]. Evidence has continued to corroborate this view. For instance, not all *PSEN1* mutations have the same predicted influence on the direction of APP processing [260]. This suggests that various mutations may not all drive disease in the same manner. Indeed, it is under-appreciated that many Aβ-independent disease mechanisms may be initiated by different autosomal dominant mutations. We provide some examples in Supplementary Material 1.5.

Although many similarities exist, autosomal dominant AD mutation carriers can have distinct pathological and clinical phenotypes, not only from the wider AD spectrum but from each other. This suggests that although they share broadly similar endpoints, they might not arrive there in the same way. Despite spatial distribution of neuropathology being similar between autosomal dominant and sporadic AD [159], Aβ pathology is more severe in autosomal dominant AD [159], and some mutation carriers can have unique plaque types. For example, individuals with *PSEN1* exon 9 deletions frequently exhibit ‘cotton wool plaques’ lacking a compact amyloid core with little neuritic and glial involvement [227]. Furthermore, Aβ42 deposition is increased in some autosomal dominant cases compared to sporadic AD [157]. In addition, recent research suggests that both autosomal dominant AD and Trisomy 21 can be differentiated from sporadic AD by a distinct pattern of early striatal and thalamic amyloid deposition [53]. On limited data, in vivo comparisons of tau pathology indicate some similarities and differences [213], but firm conclusions await more development and use of in vivo tau tracers.

Others have shown distinctly different cognitive [198, 227, 229, 247], neurological [227], metabolic [183] and biochemical [11, 200] presentations between autosomal dominant AD, early-onset AD (in general) and late-onset AD [58] (reviewed in [227, 247, 262]). Trisomy 21, too, once provided an important pillar of support for the amyloid hypothesis, but several valid findings question its relevance to all AD (refer to Supplementary Material 1.5 for extended discussion).

In summary, neither within autosomal dominant cohorts, nor between these cohorts and all AD, has either the aetiology or the aetiological relationship been determined. The phenotypic differences between autosomal dominant AD and sporadic AD raise suggestions they may have unique, or only partially overlapping, aetiologies.

### The genetic risk factors for AD are many, varied and may act through Aβ-independent mechanisms to influence disease onset and progression

The numbers of possible Aβ-independent mechanisms of AD are magnified by the many risk factors being identified through large-scale GWAS studies. To date, variants in *NOTCH3*, *MAPT*, *GRN*, *C9orf72*, *CLU*, *PICALM*, *CR1*, *MS4A4*/*MS4A6E*, *CD2AP*, *CD33*, *EPHA1*, *ABCA7*, *BIN1,* and others [105, 152, 186, 246], have been implicated. As well as evidence some of these are linked to amyloid plaque pathology [248], other studies have shown that many are unlikely to be associated with plaques and NFTs. Indeed, they even associate with other common neuropathologies [78]. For example, a recent GWAS analysis of nearly 5000 individuals illustrated only 12 of 21 risk loci for clinically-defined AD dementia were corroborated in clinico-neuropathologically defined AD brains [13].

Possible Aβ-independent disease mechanisms driven by genetic risk factors cluster in a few key pathways including cholesterol and lipid metabolism, cell adhesion pathways, immune system and inflammatory response and endocytosis (for detailed reviews see [90, 275, 278]). They also clearly associate with synaptic function: synaptic genes are sensitive to the aging process [64] and many genetic AD risk factors appear to have synaptic functions [275], including *APP* and *PSEN1*, implying synaptic pathology could be driven by perturbations to critical synaptic genes, independently of Aβ.

Of note, analysis of cell-specific expression patterns illustrated many AD-linked genes are expressed by specific cells, with microglia consistently highlighted (e.g. *TREM2* and *TYROBP* [293]). Alongside a new appreciation of the role of microglia at synapses, these findings have profoundly influenced our thinking, leading us to predict perturbations in homeostatic microglial functions at synapses, driven by many factors, could be playing a major role in AD aetiology [179, 180] (see Supplementary Material 1.3 for more information).

Several putative protective genetic factors provide clues to broader AD aetiology. One in particular has generated enormous excitement: an alanine to threonine mutation adjacent to the BACE1 cleavage site on APP [133]. Although rapidly touted as substantial support for the amyloid hypothesis (and it certainly appears so at first glance), such an interpretation fails to appreciate many alternate theories for the protective mechanism. For example, being present in both full-length APP and secreted APP alpha (sAPPα) this mutation could enhance the many synaptic, neuroprotective and neurotrophic functions of these molecules [173, 226]. Yet, this avenue has not been explored experimentally to date. As partial support for this possibility, sAPPα production trended towards an increase in carriers, and the cognition of carriers was better conserved compared with non-carriers, even after removing known AD cases from the cohort [133]. We discuss aetiological implications of other protective genetic factors, including *APOE2* and *PU.1,* in Supplementary Material 1.5.

### The genetic risk factors for AD do not all fit seamlessly within the amyloid hypothesis

Most researchers still place Aβ as central to the role of AD risk genes, despite the many possible Aβ-independent disease pathways these risk factors could be influencing. This belief heavily colours the interpretation of novel AD-associated genes. For example, the evidence base for *SORL1* to be recognised as a fourth autosomal dominant AD mutation has recently grown after finding that rare loss-of-function *SORL1* mutations are associated with a younger age-of-onset and are absent in cognitively normal populations [215]. Initial proposals regarding the pathophysiology of these variants focus almost exclusively on a potential link between SORL1 and APP processing [4, 291], despite the many ways these variants could drive disease independently of Aβ [253]. Other well-characterised genetic AD risk factors such as *TREM2* variants and *APOE4* suffer similar fates [135, 274]. For discussion of possible Aβ-independent mechanisms of *TREM2* variants and *APOE4* pathogenicity refer to Supplementary Material 1.5.

In large part the collective drive to establish Aβ-dependent mechanisms for every genetic risk factor is likely a consequence of the Aβ-lenses through which we have all thought of this disease for most our working lives. Hence, if it is first asked what a new genetic variant does to APP processing, or Aβ, before alternate functional effects are considered, Aβ-dependent, rather than Aβ-independent mechanisms, are more likely to be found. In essence, this is classical confirmation bias, born from subconscious behavioural bias, driven perhaps by funding opportunities, together herding scientists toward the analysis of only one part of the picture [7].

### A partial return to Kraepelin’s separation of early-onset AD and late-onset AD is warranted, but could now be based on genetics

The rarity and phenotypic differences of autosomal dominant AD mutations have often led to suggestions autosomal dominant AD and sporadic AD may have different aetiologies [36, 38, 252]. A split in nosology could therefore be valid, despite Katzman’s call to group early and late-onset cases together in the times before any genetic factors were identified. Others continue to emphasise the similarities as evidence they continue to be aetiologically relevant to one another [10].

It is clear that the amyloid hypothesis is most relevant to autosomal dominant AD, notwithstanding possible Aβ-independent mechanisms contributing to pathogenesis in these cohorts. Hence, clinical and pathological similarities between autosomal dominant AD and AD, generally, are important to the validity of the amyloid hypothesis in all AD. However, the idea that the amyloid hypothesis is correct for all AD is difficult to reconcile with long-standing and emerging evidence that *autosomal dominant AD can be differentiated from the far more common sporadic condition by the clinician, neuropathologist and geneticist*.

It is interesting to reflect that known autosomal dominant AD mutations may drive distinct diseases and, since autosomal dominant AD is linked to early-onset, a partial return to Kraepelin’s original distinction between presenile and senile dementia, albeit involving genetics as well as age-of-onset, could be valid. The emergence of many genetic risk factors for sporadic AD supports this further through the valid hypothesis these genetic risk factors may be driving unique pathophysiological disease mechanisms, independent of Aβ.

## Implications of Questions 1–5: the risks of labelling ‘Aβ pathology’ as ‘Alzheimer’s’

### Many findings raise questions regarding the central role of Aβ in all AD: until this is resolved, caution is needed

This kind of genuine objectivity, while seemingly a necessary quality of any scientist in his field, is systemically virtually precluded now more than 100 years later. In a modern era where science has to be sold to funding agencies in order for careers to be maintained if not advanced, where adherence to schools of thought is nakedly prejudicial, and where fealty to senior scientists has less to do with scholarship and innovation than political stratagem, it is somewhat refreshing to peruse the translated works. Moreover, Alzheimer was anything but a self-promoter, as the name of the disease that now bears his name was not put forth by Alzheimer, but his contemporary and boss at the University of Munich, Emil Kraepelin [4] (Figure 1.4). Alzheimer, for his part, went to great lengths to include the observations of others in his descriptions:

Castellani and Perry [35]

Our motive for raising questions about the position of Aβ in AD nosology, aetiology and diagnosis is not to suggest Aβ has no role in AD, nor to suggest that therapeutically targeting Aβ may not ultimately prove to have some benefit. We do not refute a role for Aβ in AD aetiology [179] and we draw no such conclusion here. We have, for example, already reviewed its role as one of the secondary damage-associated molecular pattern molecules (DAMPs) that generate proinflammatory cytokines through activating Toll-like receptors (TLRs) [48] in a number of diseases. In other words, the role of Aβ in health and disease remains an important basis of continuing research.

It is not yet known, however, exactly why Aβ accumulates in some individuals. For sporadic AD, the prevailing theory suggests failing clearance mechanisms [261], whereas in familial AD, altered proteolytic processing of APP may be the culprit, with the caveat not all autosomal dominant AD mutations have the same catalytic effect [260]. Alternatively, amyloid plaque pathology could be a *“*general type of tissue reaction*”* to a number of factors, as suggested some eight decades ago [223].

In this context, it is important to note also that although Aβ is often considered deleterious, no consensus exists yet on whether it is harmful, helpful [6, 149] or just a bystander [36, 166]. Some have suggested it is an antibacterial and antiviral [96], seals blood–brain barrier leaks and has roles in learning and memory, amongst other important physiological functions [32], some of which could explain its deposition in the aging brain [156]. As we [179] and others [32, 87, 177, 212] have previously pointed out, little is known about these roles, the physiological functions of the APP protein and other cleavage products of APP, or the physiological roles of presenilin proteins beyond APP processing. This is not to say that Aβ, or specific species of Aβ, do not also have deleterious functions, but that their role may be dynamic and more complex than toxicity alone. Further studies along these themes may help put Aβ’s role in disease in a different context.

**Fig. 2:**
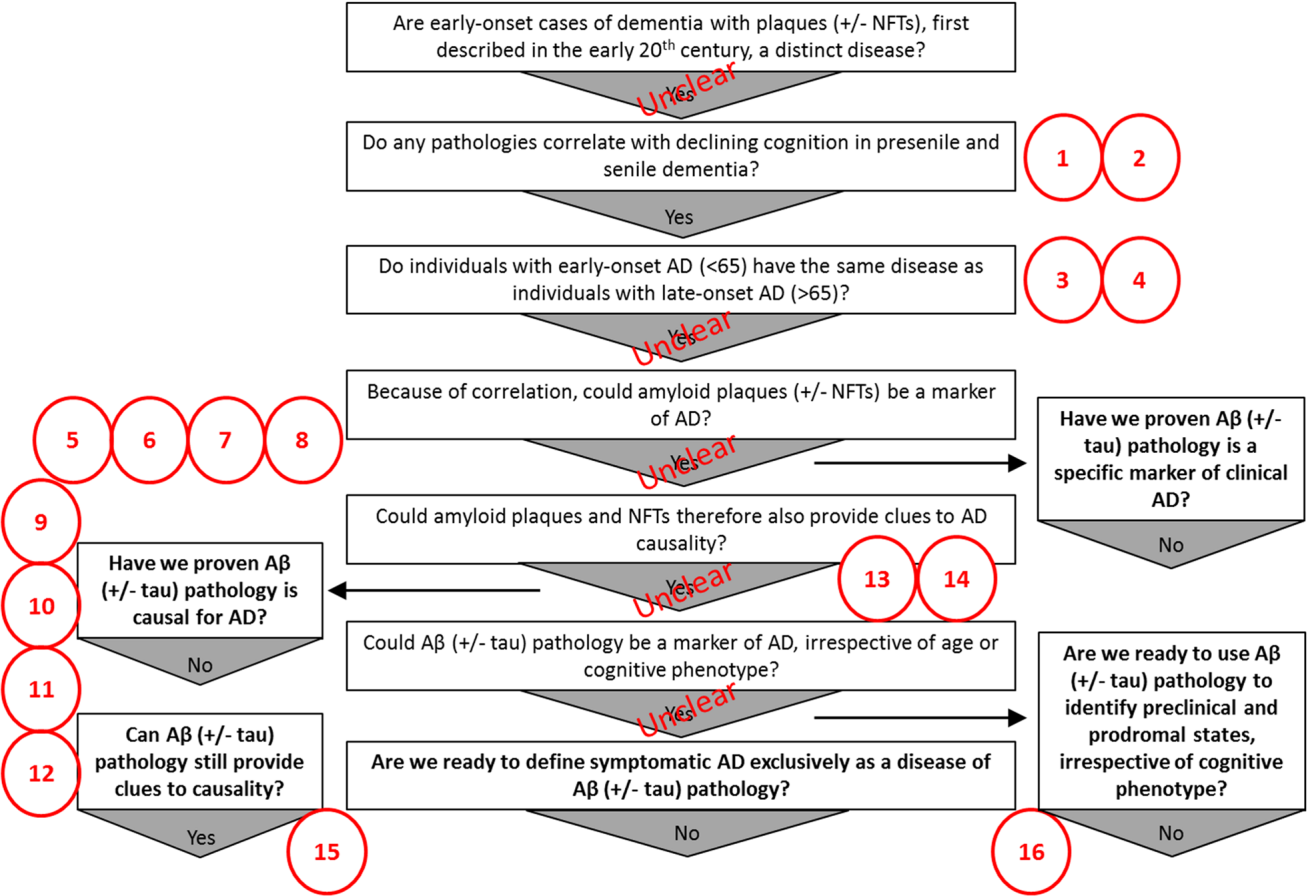
The missing pieces of the puzzle in the chronological sequence of questions, assumptions and findings that lead to the current Aβ-centric ‘consensus’ Alzheimer’s disease diagnostic guidelines. Many problems with the evidence used to support the current consensus diagnostic guidelines and the amyloid hypothesis still exist. 1. Aβ pathology does not correlate well spatially to areas undergoing atrophy. 2. Other pathologies correlate better (synapses, NFTs) but have not been as extensively studied. 3. Early-onset AD and late-onset AD might be different diseases based on evidence suggesting they can be distinguished in various ways. 4. AD may not be one single homogenous disorder. 5. Aβ pathology exists in cognitively normal individuals. 6. Aβ pathology is lacking in some with AD dementia. 7. Aβ pathology is not unique to AD dementia. 8. It is not conclusive that Aβ pathology is, temporally, the first ‘biomarker’ of AD. 9. Deleterious mutations in many genes can be linked to Aβ, but not all of them necessarily mechanistically contribute to disease through an effect on Aβ metabolism. 10. Protective environmental and genetic factors are not necessarily mechanistically protective through some effect on Aβ. 11. Much of the genetics of familial AD remains unresolved. 12. Senescence, autophagy, genetic, microbial, lifestyle choices/environmental and cardiovascular/traumatic injury risk factors are potential upstream ‘causes’ of AD, but their mechanism contribution to pathogenesis is not necessarily through Aβ. 13. Apart from genetics, most evidence supporting the amyloid hypothesis comes from preclinical research (toxicity studies, expression of human AD mutations in mice), much of which has dubious relevance to the human condition [179]. 14. Many valid alternate hypotheses of AD aetiology exist. 15. Many other possible factors independent of Aβ that could provide clues to causality exist. 16. Other methods for defining disease in preclinical, prodromal and symptomatic stages that do not require a priori stratification on the basis of Aβ measurement may exist, and remain untested. *NFTs* neurofibrillary tangles, *Aβ* amyloid-β

Irrespective of Aβ’s relevance to aetiology, there are many discrepant findings that raise doubts regarding the long-held assumption Aβ pathology *ipso facto* defines AD dementia (Fig. 2). Considering this, in the following we discuss the risks inherent in failing to adequately question the assumptions on Aβ’s position in disease and suggest ways in which disease prediction and nosology could otherwise be approached.

### Clinical practice and clinical/preclinical research heavily depend on the way AD is framed and getting it wrong has serious detrimental implications

#### Implications of the current approach for modelling of disease

The majority of preclinical models for AD have been based upon overexpression of autosomal dominant human AD mutations in mice. Criticism has been levelled at these models [48, 179, 234]. For instance, a recent article suggested up to 3000 publications may need to be re-evaluated due to overexpression artefacts [230]. Regardless of the technical issues, data generated in these models is a priori a study of autosomal dominant AD with unknown relevance to sporadic AD. Furthermore, when new genes or mechanisms are linked to AD they are invariably hypothetically considered and subsequently investigated in the context of their impact on Aβ. This represents a subconscious behavioural bias that can lead to confirmation bias [7]. Preclinical research must look beyond an Aβ-centric approach, taking cues from other AD risk factors and considering how these risk factors could mechanistically lead to cognitive loss in a truly unbiased manner.

#### Implications of the current approach for clinical research

The amyloid hypothesis has clearly held sway over the direction of interventional clinical trials [179] but this approach has so far proven unsuccessful [57, 136]. Neither has targeting of NFT deposits fared well to date [88]. Support for the amyloid hypothesis and current diagnostic guidelines for AD may well emerge from continuing clinical trials [56]. However, while any promising future results may provide impetus for pursuing such approaches further, it is important not to over-interpreting findings (Box 2). Crucially, while a positive result with an anti-Aβ agent will likely be seized upon as proof of the amyloid hypothesis, even positive outcomes will not necessarily negate the messages we relay here (see Box 2). Meanwhile, it is important to bear in mind there is an absence of evidence that modifying the levels of putative AD biomarkers, such as Aβ and tau, predicts clinical benefit. Therefore, even if such biomarkers are proven diagnostically useful, it should not be automatically assumed targeting them will provide therapeutic benefit.

For clinical research more generally, we suggest studies do not always have to be approached through an Aβ lens. It is extremely difficult to draw aetiological conclusions when experiments are designed to only include information from individuals with both a clinical and pathological diagnosis of AD, but exclude individuals with a clinical diagnosis of AD without evidence of Aβ pathology. We stress the need to retain an open mind regarding the exact aetiology of the clinical AD phenotype and to reflect this more accurately in experimental designs and therapeutic approaches.

#### Implications of the current approach for clinical practice

The way AD has been framed over the last few decades is driving clinical practice despite the fact it remains hypothetical. As a case in point, clinicians with knowledge of amyloid PET scans have changed diagnosis and treatment strategies [61], but we do not yet know if this was truly beneficial for the patients. Furthermore, the ethical dilemma of passing a diagnosis of amyloid-positivity onto patients as a diagnosis of preclinical AD, and potentially imminent AD dementia, has not been thoroughly considered. Knowledge of biomarker status may lead to stigma [258] and anxiety [97]. It is therefore important to recognise that although such biomarkers may provide some measure of risk for disease, it is not yet clear that any individual biomarker alone defines clinical AD dementia, or that targeting them will provide therapeutic benefit. We discuss possible alternate, more holistic approaches to disease prediction, diagnosis and nosology, below.

## Box 2: Possible interpretations of future clinical trials with therapeutics based on the amyloid hypothesis

1. If a therapeutic based on the amyloid hypothesis achieves a significant positive result the field may have reached the stage, predicted by Castellani and Smith [39], that eventually, by weight of numbers, a significant result is found by chance.
2. A significant positive response, perhaps on slowing the rate of progression or alleviating symptoms, will provide evidence Aβ may play some role in disease aetiology, but will not prove it has a central role (i.e. it may provide only partial support of the amyloid hypothesis). As an example, the eventual acceptance of the fact that *Helicobacter pylori*, not stomach acid, caused stomach ulcers [165], did not necessitate throwing out all the data that suggested the bystander, stomach acid, has a role. It is feasible that in a similar way, the data on Aβ does not need to be disregarded, but rather may ultimately need to be framed within a different description of disease nosology that more conclusively reflects causality (see Supplementary Materials 1.1).
3. A continuing failure of therapeutics based on the amyloid hypothesis in Phase III clinical trials will provide evidence the amyloid hypothesis is not completely correct, or that Aβ pathology is upregulated for reasons unrelated to disease progression (e.g. possibly for a protective function, such as the anti-microbial capabilities described in the text).
4. A large statistically significant and replicable effect of an anti-Aβ therapeutic in markedly slowing neurodegeneration and dementia progression, when applied to amyloid positive patients in the asymptomatic and/or prodromal stages, will provide substantial support for the amyloid hypothesis.
5. Any positive, neutral, or negative result obtained in interventional clinical trials in autosomal dominant mutation carriers may not predict outcomes in sporadic AD cohorts. Because the aetiological relationship between autosomal dominant and sporadic AD is not yet clear, the field must entertain the possibility they represent unique diseases which may ultimately need to be therapeutically approached in distinct ways.

## If not centred on Aβ, how could disease prediction and diagnosis be approached?

### A multifaceted, unbiased approach to AD prediction best reflects the current knowledge base

Prediction, diagnosis and research in autosomal dominant AD can clearly be approached differently to sporadic AD, owing to complete penetrance in autosomal dominant cases. At this stage, however, there is a lack of a clear unifying cause for sporadic AD. A unifying cause (or causes) may be found and subsequently revolutionise disease prediction and diagnosis. Emerging and exciting evidence for viral, or other microbial causes of disease, for instance, could place Aβ as a sometimes secondary factor and potentially even a protective agent [116].

Meanwhile, in the absence of a breakthrough, we envisage a future circumstance in which AD prediction strategies incorporate the wide variety of AD risk factors including senescence, autophagy, genetics, lifestyle choices/environment (education, hypertension, obesity, hearing loss, smoking, depression, physical inactivity, social isolation and diabetes [161]), or trauma (such as cardiovascular diseases (stroke and heart disease) and traumatic brain injury). In such a strategy, an aggregate score of these risk factors, adjusted for relative risk, could be obtained, but prediction would be unbiased toward any specific factor (e.g. agnostic, like the proposed ‘ATN’ system for biomarkers [118]). Biological factors, such as APOE4 or Aβ, could be incorporated into a risk matrix, but like any other factor would not be a priori required for prediction, better reflecting that AD dementia can arise in their absence and that they might be present in those not destined to develop disease.

Objective models could then be developed to determine how these risk factors converge. For example, it could be that these factors affect the penetrance of one or two key causative biological mechanisms, such as oxidative stress or neuroinflammation. It is possible convergent mechanistic links may be Aβ and tau however, as we have elaborated above, there is ample evidence to propose convergent links may also be non-Aβ/tau. Alternatively, the various risk factors may drive disease through multiple independent biological mechanisms.

It is attractive to suggest such a predictive model could help explain the clinical heterogeneity [151, 290] than pathology alone has so far, or could help to define subgroups, as previously done so using biomarker profiles [113]. Therapeutically, treatments could be tailored to the individual (i.e. moving toward precision medicine), or to subgroups, such as those with a clearly heightened risk of AD mechanistically driven primarily by inflammation, lipid metabolism, or other.

Although a multifaceted, unbiased approach to disease prediction is clearly difficult to construct and validate both financially and scientifically, evidence of the utility of more holistic predictive systems already exists. The recently developed polygenic hazard score (PHS) [63] is leading the way in this regard. Furthermore, similar personalised, label-free approaches have, unsurprisingly, already been suggested [237].

### Multifaceted predictive approaches for AD do not have to involve Aβ or tau prestratification systems

Traditional measures of pathology can still form an important part of prediction modelling, diagnosis and nosology. New research has, for instance, suggested tau filaments may adopt disease-specific folds [76] and there is much evidence that combined measures of Aβ and tau may be diagnostically useful [194], not withstanding the well-characterised clinico-neuropathological discrepancies discussed in detail above. However, seeing that it cannot yet be concluded Aβ-status accurately predicts disease onset, defines a specific disease state, or is aetiologically significant, is there any benefit to using the ‘Alzheimer’s’ name interchangeably with ‘Aβ pathology’?

One utility of stratification systems based on biomarkers is to identify cohorts of patients for specific observational and interventional trials. For instance, knowledge of Aβ status allows refinement of patient enrolment into anti-Aβ trials by removing Aβ-negative individuals who are unlikely to benefit, and whose presence may therefore create statistical noise, from the study. However, *it does not require the ‘Alzheimer’s’ label to stratify based on Aβ status*—individuals can be stratified and studied without it. Furthermore, it can be argued better, unbiased approaches to predict and define disease (as discussed above) are not being fully explored as needed, when taking this approach.

There has always been an inherent risk of using Aβ pathology to define disease. Placing any individual with ‘abnormal’ levels of Aβ under the ‘Alzheimer’s’ banner risks including individuals that may never develop AD dementia, or excluding individuals destined to develop it. For example, had in vivo biomarkers been available to indicate Sister Mary’s abnormal amyloid plaque levels in life she may have been, under the recently proposed criteria [119], a candidate for clinical AD trials, despite hindsight telling us she would never have developed cognitive decline. A more complex, unbiased and multifaceted approach to disease prediction could have identified Sister Mary’s unlikely progression.

Furthermore, applying disease labels without previously determining if these pathologies truly represent a specific disease severely limits research investigating unifying theories in which individuals with or without other certain pathologies (such as hippocampal sclerosis, tauopathy, neurodegeneration, Lewy bodies, Aβ, etc.), are, aetiologically, part of the same disease spectrum, with Aβ pathology being just one marker, albeit somewhat unreliable, for dementia risk.

With these thoughts in mind, throughout this article we have deliberating avoided, where possible, using terms such as ‘AD neuropathology’, the ‘neuropathological hallmarks of disease’, or some other variation, to refer to Aβ and/or tau pathology. Instead we have referred to them individually as ‘amyloid plaque’ or ‘Aβ’ pathology, ‘tau’ or ‘NFT’ pathology, or AD-related/associated, where appropriate. The reason should be evident: we are not yet convinced (based on current and emerging data) they alone can account for the complexity of clinical AD dementia, or are unique markers of AD dementia, particularly in the absence of symptomatic changes. The use of such terms buys into this assumption as a priori fact.

### Getting back to basics: AD is a complex cognitive disorder

The advent of genetics and imaging has taken some emphasis away from the critical need to focus on developing better detailed methods to understand normal cognition, cognitive decline and the myriad of other issues occurring alongside AD and dementia more broadly. There are clearly limitations to cognitive testing regimens for dementia [102, 104, 206], indicating more sophisticated neuropsychological assessments are required to better predict and define cognitive and other changes [21, 102, 217], particularly in preclinical stages of disease [182]. This would likely have a profound impact on predictive power [199]. In keeping with our suggestions above, the approaches used to identify and differentiate subtle early and subsequently progressive clinical phenotypes should be linked to disease risk factors using unbiased approaches.

We suggest, more broadly, that decades of advanced research in many disciplines could become better integrated with the dementia field. This would balance out the current emphasis on neuropathology. As an example, psychology research may lead to better detection of early clinical phenotypes, track progression of decline, or improvements due to intervention, and could become a major aspect of disease stratification [15].

### Disease nosology should evolve as our understanding of disease aetiology evolves

The proposals we make above suggests disease nosology may have to move away from definitions formed on the relatively simple basis of associated pathologies, unless a direct causal link of any pathology to the development of a specific clinical phenotype emerges. This is not a new suggestion. The limitations of neuropathology-based disease definitions have long been considered [37], prominently so by David Rothschild from the late 1930s onward (reviewed in [285]). Indeed, some opinions hold that much of the discrepant data suggests the current framework of AD nosology has perpetuated a myth about the true nature of the disease which could, instead, be considered as many diseases [284].

Moving forward it is imperative that AD nosology, particularly in presymptomatic stages, evolves as our understanding of disease aetiology becomes more complete, rather than in the absence of such evidence. Until then, the umbrella label ‘Alzheimer’s’ is perhaps best applied when there is symptomatic evidence of cognitive decline, rather than as a surrogate for Aβ pathology, reflecting the understanding there may be many unique pathogenic mechanisms underlying the development of AD dementia. Such a view would be better represented by an unbiased approach to prediction and diagnosis based on myriad risk factors. In this light we reiterate our full agreement with the view that the whole person and clinical picture must be considered when diagnosing, treating and researching AD [161].

## Principles to consider for AD research moving forward

The field has yet to achieve the challenge laid down by Robert Katzman in 1986; to understand the cause of disease before we can hope to prevent it [138]. We propose the following key points be critically considered and embraced going forward as the field looks to meet this challenge:

1. There is a significant body of new data in the field, much of which leads to questions surrounding the accuracy of current consensus diagnostic criteria for AD and the validity of the amyloid hypothesis supporting them. Although this does not negate the possibility that Aβ status could predict dementia risk and play some role in disease aetiology, it does question the perceived centrality of its role in all AD. Considering this, it is incorrect to perpetuate the idea Aβ causes disease or accurately defines it as a priori fact.
2. Considering the amyloid hypothesis is struggling to account for the complexity of AD, research into treatment, prediction, diagnosis and aetiology should work to incorporate the contribution of many disease risk factors in an unbiased manner. Thus, studies of humans (and preclinical models) should not solely consider Aβ-positive individuals (and, in doing so call such individuals ‘AD’), but should instead continue to include clinically identified AD cohorts irrespective of risk factors or pathology, then attempt to parse the data according to unbiased approaches.
3. The enormous effort to relate Aβ biomarkers to risk has not yet been met by studies of other possible biomarkers, such as neuroinflammation, vascular factors and synaptic/neurodegeneration markers. Future investment in longitudinal biomarker research must be more equally distributed.
4. AD dementia may, *ipso facto*, be a synaptic disorder. The field must consider the mechanistic pathways to synapse dysfunction and loss are many and varied. To prevent the risk of confirmation bias, studies looking into the mechanistic drivers underlying the contribution of AD risk factors to pathogenesis should aim to do so using unbiased approaches.
5. Results from future clinical trials will, naturally, have a major influence on research directions. We caution against over-interpretation of clinical trial data as definitive proof of the validity of any hypothesis of AD aetiology (Box 2). In particular, extrapolating results from studies in autosomal dominant AD cohorts to the wider AD spectrum must only be done with extreme caution.
6. More attention must be placed on understanding the subtleties and complexities of cognitive decline. Integrating the neuroscience and psychology of learning and memory, systems physiology, cardiovascular biology and endocrinology, immunology and more into the field could revolutionise our understanding of disease. As eloquently relayed [161], considering the person as a whole is critical to successful intervention, a view we reiterate could be extended to disease nosology, aetiology and diagnosis.

## Electronic supplementary material

Below is the link to the electronic supplementary material.

Supplementary material 1 (DOCX 147 kb)
