# Supplementary material for: Questions concerning the role of amyloid-β in the definition, aetiology and diagnosis of Alzheimer’s disease
Source: Acta Neuropathol. 2018 Oct 22;136(5):663–89. doi: 10.1007/s00401-018-1918-8 (PMC6208728; doi:10.1007/s00401-018-1918-8)
Supplement: Supplementary file 1 — Supplementary material 1 (DOCX 147 kb) [file 401_2018_1918_MOESM1_ESM.docx]

**Supplementary Materials**

**1.1 Important historical context for understanding how the diagnostic criteria for Alzheimer’s disease have reached their current point and why they may need to evolve**

***Disease nosology should ideally be built upon disease causality***

In the 20th century improved understanding of neurological disorders stimulated a push to create neurological disease definitions grounded in a biological basis. Emil Kraepelin, who named Alzheimer’s disease (AD) after his colleague’s work, was a champion of this movement [44].

It is commonly thought biologically based disease nosology hinges on known disease mechanisms and/or causative factors, but this is not always the case [65, 88, 104]. In practice, progress demands that in the absence of precise understanding of causality nosology be built upon other factors, such as symptoms of disease (i.e. an associated pathology), or a promising hypothesis of causality. Nosology based on symptoms, or on hypotheses of disease, is powerful insofar as it allows an initial framework to assist research into causality and treatment. It follows that disease definitions will remain incomplete, and subject to change, until this initial framework is either proven, or a more definitive understanding of causality emerges, at which point nosology should evolve. The example of the evolution of the nosology of tuberculosis provided in the main text illustrates this process in action.

***AD nosology is centred on the diagnostic criteria of AD, which is supported by the amyloid hypothesis***

In the case of AD dementia, our present-day nosology is built around the clinico-neuropathological guidelines for a diagnosis of AD. These guidelines are supported by a hypothesis of AD causality (the amyloid hypothesis).

The diagnostic guidelines for AD were first laid in the early 1980s, following many years of pioneering research in the late-19^th^ to mid-20^th^ centuries that identified pathological ‘lesions’ in the brain associated with age and dementia (Fig. 1, main text). Two of these, which are now known as amyloid plaques and neurofibrillary tangles (NFTs), were described by researchers before Alois Alzheimer’s 1907 publication (for recounts of this period see [8, 11, 12]). The leap in Alzheimer’s work was the identification of these neuropathological features in a case of unusually early onset, rapidly progressing dementia [11].

The hypothesis that quantification of amyloid plaques and NFTs could be used diagnostically to differentiate normal aging from dementia was supported by studies conducted by Tomlinson, Roth and Blessed from the late 1960s onward (as discussed in the main text). This idea had precedence from the work of Oskar Fischer, a contemporary of Alzheimer, who considered ‘neuritic’ plaques a possible marker of ‘senile’ (late-onset) dementia [8, 32, 35]. Alzheimer himself surmised plaques were an accompanying feature but were not necessarily the cause [3, 26]. Following the work in the 1960s however, the idea amyloid plaques and tau tangles could biologically define disease took hold and was the basis for the initial neuropathological diagnostic guidelines in the early-1980s.

***The ‘amyloid hypothesis’ emerged contemporarily with the initial neuropathological diagnostic guidelines***

Contemporarily with the formation of the initial neuropathological diagnostic guidelines for AD in the early 1980s, the peptide constituent of the amyloid plaques was characterised, further laying the foundations for what was to become known as the ‘amyloid hypothesis’ of AD.

As mentioned above, the roots of the amyloid hypothesis can be seen in the early work of Oskar Fischer (through his proposition amyloid plaques might distinguish senile dementia). However, George Glenner is often attributed as the forebear of the present day idea of Aβ being causative [38, 85] due to two seminal 1984 publications characterising the biochemical nature of amyloid plaques [33, 34]. In 1984 Glenner and Wong, referring to the amyloid peptide they named [48], stated:

*“This protein may be derived from a unique serum precursor which may provide a diagnostic test for Alzheimer's disease and a means to understand its pathogenesis.” [34]*

They were incorrect about its genesis, but their ideas Aβ could provide a clue to causality and a means for diagnosis were entrenched and significantly expanded in the works of Masters [13, 64], Selkoe [83, 84], Hardy and others [39, 41], becoming known as the ‘amyloid hypothesis’.

Thus, the amyloid hypothesis provided a theoretical framework in support of the diagnostic guidelines of AD, centred on the presence of amyloid plaques as a defining feature of disease. As elaborated in the main text, debate regarding the role of amyloid plaques in both diagnosis and aetiology continues today. This debate is central to the direction AD research will take over the coming decades.

***AD nosology must be open to change as the understanding of disease causality evolves***

To summarise the above, in the absence of a definitive understanding of causality, the present-day nosology for AD, as reflected in the diagnostic guidelines, has been built upon associated symptoms of disease (i.e. the clinical phenotype, amyloid plaques and NFTs) and a hypothesis of disease causality (the amyloid hypothesis, linking Aβ to causality). This approach has provided a useful framework for research into causality and therapy.

Importantly however, much evidence suggests this approach is struggling to cope with the complexity of AD dementia. In particular there is emerging information on potential causes of the condition beyond Aβ, suggesting the amyloid hypothesis may not be accurately describing the full picture of disease causality. Considering, as elaborated above, disease nosology should ideally be based upon causality, it follows that the nosology of AD may have to evolve to accommodate a more holistic understanding of AD causality. In doing so, this may provide a more accurate framework for approaching research and treatment.

***AD nosology has been heavily influenced by the assumption early and late-onset AD are the same disease***

An important part of any discussion on AD nosology is to note the long-standing debate regarding whether individuals with early-onset dementia have the same disease as those with late-onset dementia. The idea, suggested by Katzman in 1976 (as discussed in the main text), that early and late-onset dementia are essentially one and the same, had historical precedence in the work of Oskar Fischer in the early 20^th^ century, as well as other others [12]. In contrast to Alzheimer’s mentor Kraepelin, Fischer did not consider there to be a distinction between early and late-onset dementia [8]. Alzheimer himself may have fallen in Fischer’s camp [8, 12], despite initially remarking *“Considering everything, it seems we are dealing here with a special illness”* [2, 81]. In any case, Kraepelin's proposition that early and late-onset disease are different entities [57] was eventually lost [50], with the two coming to be viewed under the same umbrella.

As we describe in the main text, this view has been central to Aβ’s position in AD nosology as the amyloid hypothesis is strongly supported by genetic evidence from individuals with early-onset AD. In brief, some individuals with early-onset AD (although not necessarily all, see Question 5 in the main text) harbour mutations in the amyloid precursor protein (APP), presenilin 1 (PSEN1) and presenilin 2 (PSEN2), which strongly suggest altered Aβ production is a central feature of causality in these individuals. By inference, if both early and late-onset AD are considered the same disease, this suggests Aβ may be a central feature of causality in all AD. We discuss in Question 5 of the main text whether this view is still relevant in the context of the current literature.

**1.2 Extended information pertaining to Question 1: theories for how to make sense of the presence of plaques and tangles in cognitively normal individuals**

***‘Brain reserve’ and ‘cognitive reserve’***

In brief, the hypothetical concept of ‘brain reserve’ [51] suggests that cognitively normal individuals with high amyloid plaque and NFT loads might escape clinical AD essentially through possessing bigger brains and larger numbers or neurons. The related theory of ‘cognitive reserve’ suggests the cerebral processing capacity of certain individuals may be enhanced enough to compensate for high neuropathology [92] because of a higher IQ, more comprehensive education, occupational attainment and other such lifestyle factors. There is some data supporting these theories, for instance epidemiological evidence that factors such as the level of educational attainment can influence the risk of dementia [93]. There is also evidence cognitive dysfunction may be found in high amyloid non-demented individuals with more sensitive cognitive tests [79, 92]. It is indeed plausible that cognitively normal individuals with evidence of plaques and tangles after death may have had incipient disease, but did not live long enough for it to show [72].

However, the ‘reserve’ theories are not infallible: Sister Mary, for instance (described in the main text), was over a 100 years old, had neither a large brain nor high education achievement [91], suggesting factors other than cognitive reserve may have prevented her cognitive decline. Furthermore, epidemiological evidence for cognitive reserve currently lacks a neural basis to explain the association, limiting the interpretation of this theory at present [93]. Further research will be informative for the reserve theories and AD research in general. For example, determining if there are distinct neural substrates underlying cognitive reserve may be useful for predicting the risk of cognitive decline [94].

***Neuritic amyloid plaques may better predict cognitive decline than diffuse amyloid plaques***

The location or type of plaque present in high pathology controls (diffuse, dense-cored, neuritic, etc [19, 86]) might be related to pathogenesis. Sister Mary again provides a useful example having reached the Khachaturian criteria [53] due to high levels of neocortical diffuse plaques, but failing to reach the CERAD criteria [66] due to limited amounts of neocortical neuritic plaques. Therefore, fewer neocortical neuritic plaques may have spared deterioration on cognitive tasks [91] considering neuritic [99], but not diffuse [63] plaques, may be a central cause of synapse destruction in AD. Indeed, the CERAD criteria emphasizes measuring these lesions [66], although measurement of both types of plaque still form part of the NIA-AA diagnostic guidelines [46, 100]. The importance of neuritic plaques to diagnosis has been reinforced recently with the finding Thal amyloid scores are poorly predictive of cognitive decline, perhaps in part because this score does not discriminate between diffuse and neuritic plaques [87].

Nevertheless, as noted in the main text, as many as 20% of cognitively normal elderly may reach the neuropathological diagnostic criteria of AD, even when restricted to probable/definitive on the CERAD scale and intermediate/high on the NIA-Reagan scale [79]. Other studies have similarly found evidence of neuritic plaques in cognitively intact individuals, reaching varying levels of likelihood for the neuropathological diagnoses of AD [4, 10, 56], even in extreme old age [73]. In addition to neuritic plaques, Braak stages I-V exist in individuals without evidence of cognitive decline [70, 71]. Although true end-stage neuritic amyloid plaques and NFTs are rarer in the cognitively normal than those with dementia [79], end-stage lesions are also not ubiquitous in symptomatic cohorts [82].

Indeed, one researcher recently recounted “…*most neuropathologists have, however, encountered autopsy brains from cognitively intact elderly individuals that contain abundant neuritic SPs. Anecdotal reports have described all neuropathologic features of AD in cognitively normal elderly—indeed, even in rare individuals who had been examined neurologically shortly before death and judged to be cognitively intact* [102]*”.*

In summary therefore, although neuritic plaques may better correlate with disease than diffuse plaques, pathological levels of these lesions can be found in cognitively normal individuals, questioning the hypothesis pathological levels of Aβ conclusively define AD dementia as separate from normal cognitive aging. This suggests other epidemiological and biological factors may play important, as yet unrecognised roles in disease aetiology.

***Soluble Aβ oligomers, rather than insoluble plaques cause disease, which may make the presence of insoluble deposits in cognitively normal individuals irrelevant***

One persuasive explanation for the existence of high pathology controls is that plaque scores do not correlate well to dementia states because soluble Aβ oligomers, rather than insoluble species, cause synaptic and neurotoxicity in AD [40]. This hypothesis has some evidence from correlative studies [30] and a groundswell of support from many basic research efforts [85]. Quantification of oligomers could therefore be used to differentiate high pathology controls likely to develop AD on the basis of soluble, rather than insoluble levels of Aβ [15].

However, we reiterate the important caveat to this theory, noted in the main text: The oligomer theory must be tempered by the lack of consensus on the definition [9, 98], definite presence and biochemical composition [80, 103] of Aβ oligomers in the brain in vivo and the questionable validity of studies purporting oligomer toxicity, in part due to the use of non-physiologically relevant experimental paradigms [68]. As stated by Castellani and Smith, one interpretation of this situation is essentially “…that invisible molecules target invisible structures”  [20].

***Further evidence amyloid is a risk factor for disease, but is not necessarily a cause, or the primary/only cause***

Two relevant studies further corroborate the view amyloid is a risk factor for the development of cognitive decline, but not necessarily a cause, or the primary/only cause. The first illustrated the magnitude of Aβ pathology at baseline in cognitively normal individuals was linked to the magnitude of cognitive decline over 4 years [31]. The second corroborated the amyloid dose-response effect, but further suggested hippocampal atrophy and Aβ pathology have both independent and additive effects on decline in various cognitive domains [14], indicating cognitive decline in AD is not necessarily all about amyloid (for an extended commentary on these recent developments see [36]).

As we conclude in the main text, the evidence suggests pathological levels of amyloid plaques and tau tangles can be present in some cognitively normal individuals. Although there are several valid theories to explain this, in particular the reserve theories, or that these individuals may have preclinical AD but have not lived long enough to become symptomatic, these and other theories have not yet been conclusively proven. It is therefore important to continue pursuing the many other epidemiological and biological factors which may be involved in the expression or prevention of AD dementia and to incorporate these into nosology in an unbiased manner..

**1.3 Extended information pertaining to Question 3**

***Other possible explanations currently suggested for the poor correlation of Aβ pathology to cognitive decline***

- Pathology in regions without amyloid deposition, such as hypometabolism, may be caused by amyloid deposition in remote but functionally connected areas [55].
- AD might progress through synaptically connected networks, mediated by Aβ causing transsynaptic deficits when produced by entorhinal cortex neurons [42].

***Important features of cerebral amyloid angiopathy (CAA) with relevance to AD***

Some important features of CAA with relevance to AD aetiology include that CAA deposits contain a higher proportion of Aβ40 peptides than parenchymal deposits, that CAA is associated with cerebral haemorrhages and infarcts and that CAA can occur in the absence of parenchymal Aβ deposition (and vice versa) [90, 102]. Although CAA and parenchymal deposition may drive similar phenotypes, there are possible differences: CAA may drive distinct cognitive profiles [7] and although patterns of cortical thinning overlap, there are some regional differences [90].

***How might synapse dysfunction and loss theoretically occur independently of Aβ in AD?***

As but one example of how synapse dysfunction and loss may be independent of Aβ, we have previously suggested synaptic dysfunction may be driven by subtle alterations in the physiological function of microglia and other cells at the multicellular synapse [69]. Importantly, these alterations could be manifesting undetected for many decades prior to the appearance of chronic synaptic dysfunction and loss.

Dysfunction of homeostatic roles in glia could be triggered by many AD risk factors, for instance senescence [16], autophagy, genetics, microbes (bacteria, viruses, fungi etc.), lifestyle/environmental, traumatic or other causes. Notwithstanding the poor understanding and definition of the term ‘neuroinflammation’ [225], we have long [46] argued the case for a major role of proinflammatory cytokines such as TNF and IL-1 in the pathophysiology of AD upstream and downstream of amyloid and tau pathologies [48, 309]. Indeed, in a model of synapse loss, where glial cells are front and centre, cytokines could be one of the primary mechanistic drivers of glial dysfunction, downstream of the aforementioned AD risk factors. Additionally, phenomenon’s such as microglial priming [76], potentially driven by AD risk factors, could trigger heightened microglial responses later in life, exacerbating synaptic dysfunction and degeneration. More recently, support for a major role of glial dysfunction in disease has emerged in the form of evidence glial cell senescence may play a role in initiating neuropathology [16].

It is attractive to propose such a glial-centric model could conceivably extend to reversible dementia mimics, through affecting physiological synaptic functions in conditions such as depression [1] or delirium (post-surgical/alcoholism induced etc. [25, 27]), but not be followed by chronic synaptic and neuronal cell loss, explaining their transient nature. We reiterate that these mechanisms may or may not involve Aβ, so they must be investigated without bias.

In regard to the key messages for this paper, the essential point is that the field must work to develop techniques capable of detecting subtle perturbations in the physiological functions of the many molecules and cells involved in synaptic plasticity, alongside longitudinal investigations of synaptic dysfunction and loss.

**1.4 Extended information pertaining to Question 4**

***What novel in vivo markers could be used to test the hypothesis neuroinflammaton, or other disease associated pathologies, might be upstream of Aβ and predictive of cognitive decline?***

Many pathogenic mechanisms contributing to AD may be unrelated to amyloid. For example synapse damage [52] and microglial activation [77, 95] could be associated with excess TNF or IL-1 activity. In this context the capacity of these cytokines to increase the promoter activity of the *APP* gene [95] should not be ignored. However, since other DAMPs aside from Aβ can be expected to be acting (for instance S100 proteins and HMGB1 [21]), excess cytokines may or may not act independently of Aβ.

It is clearly important to consider what non-amyloid pathologies may be either upstream of amyloid, or might be independently predictive of cognitive decline in AD. Other than those discussed in the main text, promising putative markers for neuroinflammation include soluble TREM2 as a possible proxy marker of microglial activation [77, 95, 96]. In one study assessing the temporal relationship of soluble TREM2 to AD in autosomal dominant AD carriers a conclusion was made that soluble TREM2 is increased early, but after amyloid and neuronal injury changes had already occurred [95].

Microglial activation may also be measured in vivo with ligands for translocator protein 18 kD (TSPO). These have been shown to upregulate in AD in a pattern that may parallel with Aβ deposition and also correlate with scores of cognition (for a review see [43]). Furthermore, CSF measurements of YKL-40 (chitinase-3 like-1 [CHI3L1]), a putative inflammation marker secreted by astrocytes, are elevated in preclinical AD [97] and AD [24] and therefore may be able to predict cognitive decline.

In summary, many novel in vivo markers for AD-associated pathologies other than amyloid plaques and tau tangles are being developed. Investing the relationship of these markers to disease initiation and progression in an unbiased manner is an important next step.

**1.5 Extended information pertaining to Question 5**

***Statistics regarding the heritability of AD***

Before the advent of massive parallel sequencing [309] diagnostic genetic screens for AD had generally been carried out on just the Aβ coding exons in the *APP* gene, and all coding exons in the *PSEN1* and *PSEN2*, in a linear sequence [23]. Thus *PSEN1* had been screened first, followed by *APP* and *PSEN2*, reflecting the relative frequency of mutations in these genes in AD [32]. Such mutations are found almost exclusively in early-onset AD, other than in a few cases with rare coding variants that may be linked to late-onset [56, 119]. With only a few exceptions disease causing mutations in these three genes are considered completely penetrant [32].

Generally, heritability is predicted to be ~60-80% for late-onset AD and potentially 92-100% for early-onset AD [17, 105]. Early-onset AD is variably reported to represent ~1-10% of all AD [17, 108], and generally thought to be familial. However, <1% of all AD cases are currently estimated to be autosomal dominant AD [101] and up to 95% of early-onset AD cases remain genetically unexplained [17]. In one study, for instance, only a small number of early-onset AD cases (which totalled 41.2 per 100,000 people) were explained by *APP* mutations and duplications, *PSEN1* or *PSEN2* mutations (5.3 per 100,000 people [18]). Furthermore, research has shown only 4, 1.4, and 1% of patients referred for diagnostic genetic screening proved to possess mutations in *PSEN1*, *APP* and *PSEN2*, respectively [101]. Much of the genetics of AD clearly remains unresolved.

As an interesting case in point (for its historical importance in the AD field), Alzheimer’s first case, one of early-onset AD, has not yet been linked to any mutations, despite for a brief time it being thought a *PSEN1* mutation may have been involved, a finding later refuted [249].

In summary, much of the genetics of AD remains unresolved. Importantly, novel genetic linkages to disease should be investigated without bias toward what the potential pathogenic mechanism may be (we discuss this further in the main text).

***Examples of alternate pathogenic mechanisms for autosomal dominant AD mutations***

We note that none of the following examples necessarily rule out a role for Aβ in aetiology, but they illustrate how much more complex disease is than Aβ alone, even in familial cohorts.

1. Increases in BACE1 processing of APP in autosomal dominant AD carriers may be concomitant with decreased ADAM10 processing, leading to reduced levels of the putative neuroprotective molecule secreted APP alpha (sAPPα), thereby driving disease through a relative lack of a protein integral for physiological synaptic function [67].
2. Many *APP* mutations drive increases in the levels of C-terminal APP fragments. This may give rise to detrimental Aβ-independent effects on cognition by these fragments [75].
3. Mutations in *PSEN1* are the most common cause of autosomal dominant AD. PSEN1 is a protein with many substrates and therefore many integral functions independent of APP processing that could be perturbed by deleterious mutations. This view is embodied by a popular alternative hypothesis of AD aetiology, the ‘presenilin hypothesis’ [89].
4. We further discuss these issues elsewhere [68].

***How relevant is Trisomy 21 to AD?***

Trisomy 21, is often considered gold-standard evidence for the pathogenicity of Aβ. However, it is well known for its heterogeneity in the onset and presentation of disease [6, 61], despite universal deposition of Aβ (albeit with some suggestion this could be due to mosaicism [78]). Interestingly, recent research in a mouse model of Trisomy 21 has indicated genes on chromosome 21 may have a role to play in cognitive decline and amyloid deposition, independently of *APP* [106], illustrating the linear story of the amyloid hypothesis might not fully explain pathogenesis. Trisomy 21 is identified as ‘atypical dementia’ in the IWG diagnostic research criteria [29] suggesting an aetiological link between it and the wider sporadic AD condition is not definitive.

***Aetiological insights from genetic factors putatively protective against AD***

- A locus on chromosome 17, common in the general population and harbouring several chemokine genes, potentially alters the age-of-onset of MCI and dementia by ~10 years in *PSEN1* autosomal dominant AD mutation carriers, putatively through an alteration in eotaxin-1 signalling (a protein involved in neuroinflammation) [59].
- The *APOE2* genotype has been associated with ~50% reduced risk of AD, which could occur through multiple molecular mechanisms including influencing long term potentiation (LTP) and upregulation of extra cellular matrix proteins, thereby promoting neuroprotection [23].
- A polymorphism in myeloid lineage transcription factor PU.1 delays the onset of AD, possibly by affecting the expression of many AD risk genes [45]. This finding further underlines the growing important of myeloid lineage cells, such as microglia, to disease aetiology.

***Evidence supporting Aβ-independent mechanisms of pathogenesis for TREM2 variants and APOE4 in AD***

As with SORL1, discussed in the main text, both TREM2 and APOE4 have many complex physiological functions that may be perturbed (and thereby drive disease) independent of Aβ [22, 28, 107]. For instance, a recent study suggested a TREM2-APOE pathway could be a major determinant of microglial phenotypes in neurodegenerative disorders [58].

*APOE4* is, of course, particularly important to discussions of AD aetiology considering its position as the strongest genetic risk factor for sporadic AD. Under the amyloid hypothesis the consequences of *APOE4* are often seen in Aβ terms [37]. In this view one might assume the presence of *APOE4* is linked to the deposition of Aβ (i.e. in a linear sequence of APOE4 – Aβ deposition – AD dementia). But this is not always the case: although *APOE4* is often linked to heightened Aβ deposition [5], its presence does not always guarantee it. Some studies have shown no relationship between *APOE4* and cortical Aβ deposition [60] and others have surprisingly shown heightened Aβ deposition in *APOE4* non-carriers vs. carriers [62, 74].

Furthermore, pathological effects *of APOE4* may be Aβ-independent, such as links to hippocampal and cortical deformities [54] and perturbations of glucose metabolism [47]. Intriguingly, recent research has shown APOE is in the top 99% of all transcripts expressed by microglia and its expression regulates a network of other genes during aging and in response to disease [49], hinting at a role for APOE in microglial function (and, by extension, dysfunction).

Collectively, these and many other studies (beyond the scope of this review) suggest that the idea *APOE4, TREM2* variants and other disease-linked genes are risk factors for disease in a linear fashion, primarily through Aβ-dependent mechanisms, may not be wholly supported by the available evidence.

**Supplementary Materials References**

1. Alexopoulos GS, Meyers BS, Young RC, Mattis S, and Kakuma T (1993) The course of geriatric depression with "reversible dementia": a controlled study. Am J Psychiatry 150(11): 1693-9

2. Alzheimer A (1907) Ueber eine eigenartige Erkrankung der Hirnrinde. Centralblat fur Nervenheilkunde und Psychiatrie 30: 177-179

3. Alzheimer A (1991) Über eigenartige krankheitsfälle des späteren alters: (on certain peculiar diseases of old age). History of Psychiatry 2(5): 74-101

4. Andrade-Moraes CH, Oliveira-Pinto AV, Castro-Fonseca E, da Silva CG, Guimarães DM, Szczupak D et al (2013) Cell number changes in Alzheimer’s disease relate to dementia, not to plaques and tangles. Brain 136(12): 3738-3752

5. Ba M, Kong M, Li X, Ng KP, Rosa-Neto P, and Gauthier S (2016) Is ApoE ɛ 4 a good biomarker for amyloid pathology in late onset Alzheimer’s disease? Translational Neurodegeneration 5: 20

6. Ballard C, Mobley W, Hardy J, Williams G, and Corbett A (2016) Dementia in Down's syndrome. The Lancet Neurology 15(6): 622-636

7. Banerjee G, Carare R, Cordonnier C, Greenberg SM, Schneider JA, Smith EE et al (2017) The increasing impact of cerebral amyloid angiopathy: essential new insights for clinical practice. Journal of Neurology, Neurosurgery and Psychiatry 88(11): 982-994

8. Beach TG (1987) The history of Alzheimer's disease: three debates. Journal of the History of Medicine and Allied Sciences 42(3): 327-349

9. Benilova I, Karran E, and De Strooper B (2012) The toxic Aβ oligomer and Alzheimer's disease: an emperor in need of clothes. Nature Neuroscience 15: 349

10. Bennett DA, Schneider JA, Arvanitakis Z, Kelly JF, Aggarwal NT, Shah RC et al (2006) Neuropathology of older persons without cognitive impairment from two community-based studies. Neurology 66(12): 1837-44

11. Berchtold NC and Cotman CW (1998) Evolution in the Conceptualization of Dementia and Alzheimer’s Disease: Greco-Roman Period to the 1960s. Neurobiology of Aging 19(3): 173-189

12. Berrios G (1990) Alzheimer's disease: A conceptual history. International Journal of Geriatric Psychiatry 5(6): 355-365

13. Beyreuther K and Masters CL (1991) Amyloid precursor protein (APP) and beta A4 amyloid in the etiology of Alzheimer's disease: precursor-product relationships in the derangement of neuronal function. Brain Pathology 1(4): 241-51

14. Bilgel M, An Y, Helphrey J, Elkins W, Gomez G, Wong DF et al (2018) Effects of amyloid pathology and neurodegeneration on cognitive change in cognitively normal adults. Brain 141(8): 2475-2485

15. Bilousova T, Miller CA, Poon WW, Vinters HV, Corrada M, Kawas C et al (2016) Synaptic amyloid-β oligomers precede p-Tau and differentiate high pathology control cases. The American Journal of Pathology 186(1): 185-198

16. Bussian TJ, Aziz A, Meyer CF, Swenson BL, van Deursen JM, and Baker DJ (2018) Clearance of senescent glial cells prevents tau-dependent pathology and cognitive decline. Nature

17. Cacace R, Sleegers K, and Van Broeckhoven C (2016) Molecular genetics of early-onset Alzheimer's disease revisited. Alzheimer's & Dementia 12(6): 733-748

18. Campion D, Dumanchin C, Hannequin D, Dubois B, Belliard S, Puel M et al (1999) Early-onset autosomal dominant Alzheimer disease: prevalence, genetic heterogeneity, and mutation spectrum. American Journal of Human Genetics 65(3): 664-670

19. Castellani R and Perry G (2013) Molecular Pathology of Alzheimer's Disease. Biota Publishing.

20. Castellani RJ and Smith MA (2011) Compounding artefacts with uncertainty, and an amyloid cascade hypothesis that is 'too big to fail'. J Pathol 224(2): 147-52

21. Clark IA and Vissel B (2015) Amyloid beta: one of three danger-associated molecules that are secondary inducers of the proinflammatory cytokines that mediate Alzheimer's disease. Br J Pharmacol 172(15): 3714-27

22. Condello C, Yuan P, and Grutzendler J (2018) Microglia-Mediated Neuroprotection, TREM2, and Alzheimer’s Disease: Evidence From Optical Imaging. Biological Psychiatry 83(4): 377-387

23. Conejero-Goldberg C, Gomar JJ, Bobes-Bascaran T, Hyde TM, Kleinman JE, Herman MM et al (2014) APOE2 enhances neuroprotection against Alzheimer’s disease through multiple molecular mechanisms. Molecular Psychiatry 19: 1243

24. Craig-Schapiro R, Perrin RJ, Roe CM, Xiong C, Carter D, Cairns NJ et al (2010) YKL-40: A novel prognostic fluid biomarker for preclinical Alzheimer’s disease. Biological psychiatry 68(10): 903-912

25. Cunningham C (2011) Systemic inflammation and delirium – important co-factors in the progression of dementia. Biochemical Society transactions 39(4): 945-953

26. Davis Ii JN and Chisholm JC (1999) Alois Alzheimer and the amyloid debate. Nature 400: 810

27. de la Torre JC (2016) Masquerading as Dementia, in Alzheimer’s Turning Point: A Vascular Approach to Clinical Prevention. Springer International Publishing.

28. DiBattista AM, Dumanis SB, Newman J, and Rebeck GW (2016) Identification and modification of amyloid-independent phenotypes of APOE4 mice. Experimental neurology 280: 97-105

29. Dubois B, Feldman HH, Jacova C, Hampel H, Molinuevo JL, Blennow K et al (2014) Advancing research diagnostic criteria for Alzheimer's disease: the IWG-2 criteria. Lancet Neurol 13(6): 614-29

30. Esparza TJ, Zhao H, Cirrito JR, Cairns NJ, Bateman RJ, Holtzman DM et al (2013) Amyloid-beta oligomerization in Alzheimer dementia vs. high pathology controls. Annals of neurology 73(1): 104-119

31. Farrell ME, Kennedy KM, Rodrigue KM, Wig G, Bischof GN, Rieck JR et al (2017) Association of longitudinal cognitive decline with amyloid burden in middle-aged and older adults: evidence for a dose-response relationship. JAMA Neurol 74(7): 830-838

32. Fischer O (1907) Miliare nekrosen mit drusigen wucherungen der neurofibrillen, eine regelmässige veränderung der hirnrinde bei seniler demenz. European Neurology 22(4): 361-372

33. Glenner GG and Wong CW (1984) Alzheimer's disease and Down's syndrome: Sharing of a unique cerebrovascular amyloid fibril protein. Biochemical and Biophysical Research Communications 122(3): 1131-1135

34. Glenner GG and Wong CW (1984) Alzheimer's disease: initial report of the purification and characterization of a novel cerebrovascular amyloid protein. Biochem Biophys Res Commun 120(3): 885-90

35. Goedert M (2009) Oskar Fischer and the study of dementia. Brain 132(4): 1102-1111

36. Gonneaud J and Chételat G (2018) Which is to blame for cognitive decline in ageing: amyloid deposition, neurodegeneration or both? Brain 141(8): 2237-2241

37. Hanon O, Vidal J-S, Lehmann S, Bombois S, Allinquant B, Tréluyer JM et al (2018) Plasma amyloid levels within the Alzheimer's process and correlations with central biomarkers. Alzheimer's & Dementia 14(7): 858-868

38. Hardy J (2017) The discovery of Alzheimer‐causing mutations in the APP gene and the formulation of the “amyloid cascade hypothesis”. The FEBS Journal 284(7): 1040-1044

39. Hardy J and Allsop D (1991) Amyloid deposition as the central event in the aetiology of Alzheimer's disease. Trends in Pharmacological Sciences 12: 383-388

40. Hardy J and Selkoe DJ (2002) The amyloid hypothesis of Alzheimer's disease: progress and problems on the road to therapeutics. Science 297(5580): 353-356

41. Hardy JA and Higgins GA (1992) Alzheimer's disease: the amyloid cascade hypothesis. Science 256(5054): 184-5

42. Harris JA, Devidze N, Verret L, Ho K, Halabisky B, Thwin MT et al (2010) Transsynaptic progression of amyloid-β-induced neuronal dysfunction within the entorhinal-hippocampal network. Neuron 68(3): 428-441

43. Heneka MT, Carson MJ, El Khoury J, Landreth GE, Brosseron F, Feinstein DL et al (2015) Neuroinflammation in Alzheimer's Disease. The Lancet. Neurology 14(4): 388-405

44. Hoff P (2015) The Kraepelinian tradition. Dialogues in Clinical Neuroscience 17(1): 31-41

45. Huang KL, Marcora E, Pimenova AA, Di Narzo AF, Kapoor M, Jin SC et al (2017) A common haplotype lowers PU.1 expression in myeloid cells and delays onset of Alzheimer's disease. Nat Neurosci 20(8): 1052-1061

46. Hyman BT, Phelps CH, Beach TG, Bigio EH, Cairns NJ, Carrillo MC et al (2012) National Institute on Aging–Alzheimer’s Association guidelines for the neuropathologic assessment of Alzheimer’s disease. Alzheimer's & Dementia 8(1): 1-13

47. Jagust WJ and Landau SM (2012) Apolipoprotein E, not fibrillar β-amyloid, reduces cerebral glucose metabolism in normal aging. The Journal of Neuroscience 32(50): 18227-18233

48. Jucker M, Beyreuther K, Haass C, and Nitsch RM (2006) Alzheimer: 100 Years and Beyond. Springer Science & Business Media.

49. Kang SS, Ebbert MTW, Baker KE, Cook C, Wang X, Sens JP et al (2018) Microglial translational profiling reveals a convergent APOE pathway from aging, amyloid, and tau. The Journal of Experimental Medicine 215(9): 2235-2245

50. Katzman R (1976) The prevalence and malignancy of alzheimer disease: A major killer. Archives of Neurology 33(4): 217-218

51. Katzman R, Terry R, DeTeresa R, Brown T, Davies P, Fuld P et al (1988) Clinical, pathological, and neurochemical changes in dementia: a subgroup with preserved mental status and numerous neocortical plaques. Ann Neurol 23(2): 138-44

52. Kester MI, Teunissen CE, Crimmins DL, Herries EM, Ladenson JH, Scheltens P et al (2015) Neurogranin as a cerebrospinal fluid biomarker for synaptic loss in symptomatic Alzheimer disease. JAMA neurology 72(11): 1275-1280

53. Khachaturian ZS (1985) Diagnosis of Alzheimer's disease. Arch Neurol 42(11): 1097-105

54. Kim YJ, Cho H, Kim YJ, Ki CS, Chung SJ, Ye BS et al (2015) Apolipoprotein e4 affects topographical changes in hippocampal and cortical atrophy in Alzheimer's disease dementia: a five-year longitudinal study. J Alzheimers Dis 44(4): 1075-85

55. Klupp E, Grimmer T, Tahmasian M, Sorg C, Yakushev I, Yousefi BH et al (2015) Prefrontal hypometabolism in Alzheimer disease Is related to longitudinal amyloid accumulation in remote brain regions. Journal of Nuclear Medicine 56(3): 399-404

56. Knopman DS, Parisi JE, Salviati A, Floriach-Robert M, Boeve BF, Ivnik RJ et al (2003) Neuropathology of cognitively normal elderly. J Neuropathol Exp Neurol 62(11): 1087-95

57. Kraepelin E (1909-1915) Psychiatrie: Ein Lehrbuch für Studirende und Aerzte, 8th ed. Leipzig, Barth.

58. Krasemann S, Madore C, Cialic R, Baufeld C, Calcagno N, El Fatimy R et al (2017) The TREM2-APOE pathway drives the transcriptional phenotype of dysfunctional microglia in neurodegenerative diseases. Immunity 47(3): 566-581.e9

59. Lalli MA, Bettcher BM, Arcila ML, Garcia G, Guzman C, Madrigal L et al (2015) Whole-genome sequencing suggests a chemokine gene cluster that modifies age at onset in familial Alzheimer's disease. Molecular Psychiatry 20: 1294

60. Landén M, Thorsell A, Wallin A, and Blennow K (1996) The apolipoprotein E allele epsilon 4 does not correlate with the number of senile plaques or neurofibrillary tangles in patients with Alzheimer's disease. Journal of Neurology, Neurosurgery, and Psychiatry 61(4): 352-356

61. Lautarescu BA, Holland AJ, and Zaman SH (2017) The early presentation of dementia in people with Down syndrome: a systematic review of longitudinal studies. Neuropsychology Review 27(1): 31-45

62. Lehmann M, Ghosh PM, Madison C, Karydas A, Coppola G, O’Neil JP et al (2014) Greater medial temporal hypometabolism and lower cortical amyloid burden in ApoE4-positive AD patients. Journal of neurology, neurosurgery, and psychiatry 85(3): 266-273

63. Masliah E, Terry RD, Mallory M, Alford M, and Hansen LA (1990) Diffuse plaques do not accentuate synapse loss in Alzheimer's disease. The American Journal of Pathology 137(6): 1293-1297

64. Masters CL and Selkoe DJ (2012) Biochemistry of amyloid β-protein and amyloid deposits in Alzheimer disease. Cold Spring Harbor Perspectives in Medicine 2(6): a006262

65. Maung HH (2017) The causal explanatory functions of medical diagnoses. Theoretical Medicine and Bioethics 38(1): 41-59

66. Mirra SS, Heyman A, McKeel D, Sumi SM, Crain BJ, Brownlee LM et al (1991) The Consortium to Establish a Registry for Alzheimer's Disease (CERAD) Part II. Standardization of the neuropathologic assessment of Alzheimer's disease. Neurology 41(4): 479-479

67. Mockett BG, Richter M, Abraham WC, and Müller UC (2017) Therapeutic potential of secreted amyloid precursor protein APPsα. Frontiers in Molecular Neuroscience 10: 30

68. Morris GP, Clark IA, and Vissel B (2014) Inconsistencies and controversies surrounding the Amyloid Hypothesis of Alzheimer's disease. Acta Neuropathol Commun 2(1): 135

69. Morris GP, Clark IA, Zinn R, and Vissel B (2013) Microglia: A new frontier for synaptic plasticity, learning and memory, and neurodegenerative disease research. Neurobiology of Learning and Memory 105: 40-53

70. Mufson EJ, Malek-Ahmadi M, Perez SE, and Chen K (2016) Braak staging, plaque pathology and APOE status in elderly persons without cognitive impairment. Neurobiology of aging 37: 147-153

71. Mufson EJ, Malek-Ahmadi M, Snyder N, Ausdemore J, Chen K, and Perez SE (2016) Braak stage and trajectory of cognitive decline in non-cognitively impaired elders. Neurobiology of aging 43: 101-110

72. Nelson PT, Braak H, and Markesbery WR (2009) Neuropathology and cognitive impairment in Alzheimer disease: a complex but coherent relationship. Journal of neuropathology and experimental neurology 68(1): 1-14

73. Neltner JH, Abner EL, Jicha GA, Schmitt FA, Patel E, Poon LW et al (2016) Brain pathologies in extreme old age. Neurobiology of aging 37: 1-11

74. Ossenkoppele R, van der Flier WM, Zwan MD, Adriaanse SF, Boellaard R, Windhorst AD et al (2013) Differential effect of APOE genotype on amyloid load and glucose metabolism in AD dementia. Neurology 80(4): 359-365

75. Pera M, Larrea D, Guardia‐Laguarta C, Montesinos J, Velasco KR, Agrawal RR et al (2017) Increased localization of APP‐C99 in mitochondria‐associated ER membranes causes mitochondrial dysfunction in Alzheimer disease. The EMBO Journal 36(22): 3356-3371

76. Perry VH and Holmes C (2014) Microglial priming in neurodegenerative disease. Nature Reviews Neurology 10: 217

77. Piccio L, Deming Y, Del-Águila JL, Ghezzi L, Holtzman DM, Fagan AM et al (2016) Cerebrospinal fluid soluble TREM2 is higher in Alzheimer disease and associated with mutation status. Acta neuropathologica 131(6): 925-933

78. Potter H (2016) Beyond Trisomy 21: phenotypic variability in people with Down Syndrome explained by further chromosome mis-segregation and mosaic aneuploidy. Journal of Down Syndrome & chromosome abnormalities 2(1): 109

79. Price JL, McKeel DW, Buckles VD, Roe CM, Xiong C, Grundman M et al (2009) Neuropathology of nondemented aging: presumptive evidence for preclinical Alzheimer disease. Neurobiology of aging 30(7): 1026-1036

80. Pujol-Pina R, Vilaprinyó-Pascual S, Mazzucato R, Arcella A, Vilaseca M, Orozco M et al (2015) SDS-PAGE analysis of Aβ oligomers is disserving research into Alzheimer´s disease: appealing for ESI-IM-MS. Scientific Reports 5: 14809

81. Rainulf S, Schnitzlein N, and Murtagh R (1995) An english translation of alzheimer's 1907 paper, “über eine eigenartige erkankung der hirnrinde”. Clinical Anatomy 8(6): 429-431

82. SantaCruz KS, Sonnen JA, Pezhouh MK, Desrosiers MF, Nelson PT, and Tyas SL (2011) Alzheimer disease pathology in subjects without dementia in two studies of aging: the Nun Study and the Adult Changes in Thought Study. Journal of neuropathology and experimental neurology 70(10): 832-840

83. Selkoe DJ (1989) Amyloid beta protein precursor and the pathogenesis of Alzheimer's disease. Cell 58(4): 611-2

84. Selkoe DJ (1991) The molecular pathology of Alzheimer's disease. Neuron 6(4): 487-498

85. Selkoe DJ and Hardy J (2016) The amyloid hypothesis of Alzheimer's disease at 25 years. EMBO Molecular Medicine 8(6): 595-608

86. Serrano-Pozo A, Frosch MP, Masliah E, and Hyman BT (2011) Neuropathological Alterations in Alzheimer Disease. Cold Spring Harbor Perspectives in Medicine: 1(1): a006189

87. Serrano-Pozo A, Qian J, Muzikansky A, Monsell SE, Montine TJ, Frosch MP et al (2016) Thal amyloid stages do not significantly impact the correlation between neuropathological change and cognition in the Alzheimer disease continuum. Journal of Neuropathology & Experimental Neurology 75(6): 516-526

88. Severinsen M (2001) Principles behind definitions of diseases--a criticism of the principle of disease mechanism and the development of a pragmatic alternative. Theor Med Bioeth 22(4): 319-36

89. Shen J and Kelleher RJ, 3rd (2007) The presenilin hypothesis of Alzheimer's disease: evidence for a loss-of-function pathogenic mechanism. Proc Natl Acad Sci U S A 104(2): 403-9

90. Smith EE (2018) Cerebral amyloid angiopathy as a cause of neurodegeneration. Journal of Neurochemistry 144(5): 651-658

91. Snowdon DA (1997) Aging and Alzheimer's disease: lessons from the Nun Study. Gerontologist 37(2): 150-6

92. Stern Y (2002) What is cognitive reserve? Theory and research application of the reserve concept. J Int Neuropsychol Soc 8(3): 448-60

93. Stern Y (2012) Cognitive reserve in ageing and Alzheimer's disease. Lancet neurology 11(11): 1006-1012

94. Stites SD, Milne R, and Karlawish J (2018) Advances in Alzheimer's imaging are changing the experience of Alzheimer's disease. Alzheimer's & Dementia: Diagnosis, Assessment & Disease Monitoring 10: 285-300

95. Suárez-Calvet M, Araque Caballero MÁ, Kleinberger G, Bateman RJ, Fagan AM, Morris JC et al (2016) Early changes in CSF sTREM2 in dominantly inherited Alzheimer’s disease occur after amyloid deposition and neuronal injury. Science Translational Medicine 8(369): 369ra178-369ra178

96. Suárez‐Calvet M, Kleinberger G, Araque Caballero MÁ, Brendel M, Rominger A, Alcolea D et al (2016) sTREM2 cerebrospinal fluid levels are a potential biomarker for microglia activity in early‐stage Alzheimer's disease and associate with neuronal injury markers. EMBO Molecular Medicine 8(5): 466-476

97. Sutphen CL, Jasielec MS, Shah AR, Macy EM, Xiong C, Vlassenko AG et al (2015) Longitudinal cerebrospinal fluid biomarker changes in preclinical Alzheimer disease during middle age. JAMA neurology 72(9): 1029-1042

98. Teplow DB (2013) On the subject of rigor in the study of amyloid β-protein assembly. Alzheimer's Research & Therapy 5(4): 39-39

99. Terry RD, Masliah E, Salmon DP, Butters N, DeTeresa R, Hill R et al (1991) Physical basis of cognitive alterations in alzheimer's disease: Synapse loss is the major correlate of cognitive impairment. Annals of Neurology 30(4): 572-580

100. Thal DR, Rüb U, Schultz C, Sassin I, Ghebremedhin E, Del Tredici K et al (2000) Sequence of Aβ-protein deposition in the human medial temporal lobe. Journal of Neuropathology & Experimental Neurology 59(8): 733-748

101. Van Cauwenberghe C, Van Broeckhoven C, and Sleegers K (2015) The genetic landscape of Alzheimer disease: clinical implications and perspectives. Genetics In Medicine 18: 421

102. Vinters HV (2015) Emerging concepts in Alzheimer's disease. Annual Review of Pathology: Mechanisms of Disease 10(1): 291-319

103. Watt AD, Perez KA, Rembach A, Sherrat NA, Hung LW, Johanssen T et al (2013) Oligomers, fact or artefact? SDS-PAGE induces dimerization of beta-amyloid in human brain samples. Acta Neuropathol 125(4): 549-64

104. Whitbeck C (1981) What is diagnosis? Some critical reflections. Metamedicine 2(3): 319-329

105. Wingo TS, Lah JJ, Levey AI, and Cutler DJ (2012) Autosomal recessive causes likely in early-onset Alzheimer disease. Archives of Neurology 69(1): 59-64

106. Wiseman FK, Pulford LJ, Barkus C, Liao F, Portelius E, Webb R et al (2018) Trisomy of human chromosome 21 enhances amyloid-β deposition independently of an extra copy of APP. Brain 141(8): 2457-2474

107. Wolf AB, Valla J, Bu G, Kim J, LaDu MJ, Reiman EM et al (2013) Apolipoprotein E as a β-amyloid-independent factor in Alzheimer’s disease. Alzheimer's Research & Therapy 5(5): 38-38

108. Zhu X-C, Tan L, Wang H-F, Jiang T, Cao L, Wang C et al (2015) Rate of early onset Alzheimer’s disease: a systematic review and meta-analysis. Annals of Translational Medicine 3(3): 38
